# Reusable and Soft Self-Adhesive Epidermal Electrodes for Human Skin Enabled by Functional Additives

**DOI:** 10.3390/mi17091066

**Published:** 2026-09-08

**Authors:** Sungmin Bae, Dong-Jin Lee, Chuljin Hwang, Dae Yu Kim

**Affiliations:** 1 Department of Electrical and Electronic Engineering, College of Engineering, Inha University, Incheon 22212, Republic of Korea; youaresm@inha.edu; 2 Center for Sensor Systems, Inha University, Incheon 22212, Republic of Korea; djlee@inha.ac.kr (D.-J.L.); aki@inha.ac.kr (C.H.); 3 Department of Electrical and Computer Engineering, College of Engineering, Inha University, Incheon 22212, Republic of Korea; 4 Inha Research Institute for Aerospace Medicine, Inha University, Incheon 22212, Republic of Korea

**Keywords:** epidermal electrodes, PEDOT:PSS, self-adhesive, biopotential sensing, electrocardiography, electromyography, stretchable electronics

## Abstract

Wearable electronics, particularly dry epidermal electrodes, provide human-connected interfaces for recording biopotential signals. However, their practical utility is often hindered by their limited operational longevity and the resulting environmental burden of electronic waste, as most conventional electrodes are discarded after a single use because of performance degradation. Herein, a reusable, soft, and conductive epidermal electrode is reported, fabricated through the precise incorporation of functional additives. By intentionally modulating the polymer chain architecture, a homogeneous composite is developed that exhibits exceptional flexibility, high conductivity (~100 S/cm), softness (~649 kPa), and stretchability (~234%). This molecular-level design promotes strong intermolecular interactions at the skin–electrode interface, facilitating persistent adhesion and conformability to challenging surfaces, including wet, wrinkled, and stretched skin. These properties enable reliable electrocardiography acquisition through 50 repeated attachment and detachment cycles, over which a commercial Ag/AgCl gel electrode became unmeasurable after 20. The applicability of the electrode to human–machine interfaces is further demonstrated by capturing clear electromyography signals of muscle activity during a rock–paper–scissors game. This low-modulus electrode platform offers a route towards repeated-use wearable healthcare systems and soft-robotics applications, with the potential to reduce the waste associated with single-use electrodes.

## 1. Introduction

The emergence of epidermal electronics has ushered in a new era of continuous healthcare monitoring by establishing a seamless interface between electronic devices and human skin [1,2]. To achieve high-fidelity acquisition of biopotential signals, such as electrocardiography (ECG) [3,4] and electromyography (EMG) [5,6,7], epidermal electrodes must satisfy a demanding set of requirements: high electrical conductivity for clear signal transmission, superior stretchability to withstand skin deformation, and crucially, a low Young’s modulus to ensure stable, long-term integration with human soft tissue without mechanical mismatch [8,9,10].

Although substantial progress has been made in this field [11,12,13,14], the practical application of epidermal electrodes is significantly hampered by their limited lifespan and the resulting environmental impact [15,16,17]. Conventional Ag/AgCl gel electrodes are predominantly single-use [18] because their conductive gels dehydrate and their adhesive properties fail [19,20] after a single detachment, leading to massive electronic waste (e-waste) and rising healthcare costs. Although recent developments in dry electrodes have successfully integrated conductivity with stretchability [21,22,23], they often struggle to maintain stable adhesion over repeated attachment/detachment cycles. Notably, the Young’s moduli of in vitro and in vivo epidermis have been reported to be approximately 1 MPa and 4 MPa [24,25], respectively, making mechanical matching with these biological values crucial for stable conformal skin contact. Nevertheless, most existing dry electrodes remain limited by their much higher Young’s moduli (>4 MPa) [21,22,23], which lead to mechanical mismatch and delamination [10,23].

The challenges lie in developing an electrode platform that simultaneously combines high conductivity, extreme stretchability, and a tissue-like low modulus while ensuring retainable adhesiveness for multiple uses. This inherent difficulty stems from fundamentally conflicting molecular mechanisms: high electrical conductivity relies on a continuous, dense percolation network of rigid-conductive pathways [26,27,28], which severely restricts polymer chain mobility and increases Young’s modulus. Conversely, achieving tissue-like softness and high stretchability requires loosely crosslinked, highly flexible elastomeric networks [29], which inevitably dilute and disrupt conductive pathways. Furthermore, securing persistent self-adhesiveness requires a delicate balance between interfacial viscoelastic dissipation for initial tackiness and high bulk cohesive strength for reusability, properties that often conflict with one another under cyclic mechanical peeling [30]. Owing to these mutually exclusive molecular criteria, the simultaneous optimization of all four parameters introduces severe material-level tradeoffs. For example, a recently reported PEDOT:PSS/PVA electrode [31] featuring a low Young’s modulus (~404 kPa), high stretchability (~157%), and good adhesiveness (~0.51 N/cm) suffers from poor electrical conductivity (<10 S/cm). Conversely, the PEDOT:PSS/WPU electrode [23] with a high conductivity (~400 S/cm) and a high adhesiveness (~0.43 N/cm) is limited by its relatively low stretchability (~50%) and high Young’s modulus (~30 MPa). To overcome these limitations, conventional strategies have adopted various molecular additives such as maltitol [10], d-sorbitol [23] and tea polyphenol (TP) [31] to modulate Young’s modulus, stretchability, and adhesiveness, respectively. Although these strategies are feasible, the addition of chemical modifiers often degrades the electrical conductivity and subsequent signal quality because of the reduced volume fraction of the conductive components [32].

In addition, interest in epidermal electrodes and flexible sensor arrays is driven partly by downstream data processing, where artificial intelligence and data-driven state estimation are now widely applied. For instance, a fine-tuned multimodal large language model (LLM) was recently used for autonomous state cognition in a shape-recognizing six-bar tensegrity structure with integrated flexible sensor networks [33]. Similarly, machine-learning decoding algorithms have been paired with stretchable sensor arrays to recognize gestures, postures, and biopotential signals [34,35,36]. These systems require electrodes that maintain low, stable skin–electrode impedance and conformal, robust contact over long and repeated recording sessions. If the signal drifts or contact degrades, the recorded data shift away from the model’s training distribution, reducing decision accuracy regardless of model design. Long-term interfacial stability is therefore not merely a matter of user comfort—it directly limits the performance of the entire sensing-to-decision pipeline.

In this paper, we report a highly reusable, soft, conductive, and stretchable epidermal electrode that overcomes these fundamental tradeoffs. A widely used conductive polymer, poly(3,4-ethylenedioxythiophene) polystyrene sulfonate (PEDOT:PSS), was emulsified using an elastomer matrix, polydimethylsiloxane (PDMS), to form a soft composite. Incorporating a small amount of poly(ethyleneimine) ethoxylate (PEIE) produces a homogeneous composite structure and lowers the Young’s modulus without altering the elastomer ratio, leading to high stretchability and softness without compromising electrical conductivity. Specifically, the PEIE loading modulates the hydrosilylation of PDMS, yielding highly flexible siloxane chains. These internally tailored chains reinforce the intermolecular attractions with the skin, such as van der Waals forces, by maximizing the effective skin contact area. This molecular design enables the electrode to achieve low skin-contact impedance (53 kΩ cm^2^ at 10 Hz) even on wet skin, facilitating accurate ECG monitoring even after 50 attachment/detachment cycles with significantly lower root-mean-square (RMS) noise (~16 μV) than commercial Ag/AgCl gel electrodes. Furthermore, we demonstrate the electrode in a human–machine interface (HMI) context, recording EMG signals during a rock–paper–scissors game in which the three hand gestures gave distinguishable signal amplitudes.

## 2. Materials and Methods

### 2.1. Materials

PEDOT:PSS (Clevios PH 1000) and PDMS (Sylgard 184) were purchased from Heraeus (Hanau, Germany) and Dow Corning (Midland, MI, USA), respectively. PEIE (37 wt% in H_2_O) and Tween 80 were obtained from Sigma-Aldrich (St. Louis, MO, USA). Ethylene glycol (EG) was purchased from Sigma-Aldrich and used to maximize the conductivity of PEDOT:PSS.

### 2.2. Fabrication of PDE Films

The fabrication of reusable and soft films began with a 2× concentration of PEDOT:PSS solution. Pristine PEDOT:PSS solutions were stirred at 50 °C and 300 rpm for volatilization of the water until the desired mass was achieved. EG solution, 10 wt% of the total mass of PEDOT:PSS, was added, and the solution was vortexed until fully dispersed. Before mixing the PEDOT:PSS/EG solutions with PDMS, 6.5 g of PDMS [10:1 (*w*/*w*) base/curing agent] and various loadings of PEIE were added to the prepared containers and blended at 2000 rpm for 30 min using a conditioning mixer. Then, 3.5 g of the PEDOT:PSS/EG solution and 0.3 g of Tween 80 solution were loaded into the pre-blended PDMS/PEIE solutions and rigorously mixed with a conditioning mixer (2000 rpm for 60 min). After the emulsified blends were fully debubbled under vacuum, they were bar-bladed onto customized molds. The resulting blend solutions were cured on a hot plate at 100 °C for 2–3 h, yielding films with an approximate thickness of 300 μm.

### 2.3. Characterization

Cross-sectional and surface images of the reusable and soft films were captured using field-emission scanning electron microscopy (FE-SEM; Model S-4300SE, Hitachi Co., Ltd., Tokyo, Japan). The average size of the PDMS cores and the full width at half maximum (FWHM) of the brightness were obtained from the SEM images using ImageJ (version 1.46r). A Fourier transform infrared vacuum spectrometer (FT-IR; Model VERTEX 80V, Bruker Co., Ltd., Ettlingen, Germany) was used to obtain the absorbance peaks of Si–O–Si and Si–CH_3_ bonds in PDMS.

The mechanical properties of the reusable and soft films were characterized using a tensile tester (Model EasyMESUR F105, Mark-10 Corp., Copiague, NY, USA) and film gripper (G1008, Mark-10 Corp.). All stretch/release cycles were performed at a 50 mm/min strain rate. Adhesion was measured by 90° peel at a displacement rate of 1000 mm/min. We chose this rate to match the rates used in the epidermal-electrode studies against which our values are compared, 1000 mm/min in Ref. [37] and 1200 mm/min in Ref. [28]. It is higher than the 300 mm/min specified in ASTM D3330 [38], so the absolute values reported here are not directly comparable with data acquired under that standard. A four-point probe tester (Model HPS2526, HELPASS Corp., Changzhou, China) was used to measure the conductivities of the reusable and soft films. To further characterize the normalized resistance of the reusable and soft films, a source meter unit (Model Keithley 2400, Tektronix, Inc., Beaverton, OR, USA) and a tensile tester were used concurrently.

For adhesion testing, PDE composite films were cut into standardized strips of 1 cm × 5 cm. Prior to testing, the skin site was cleansed with isopropyl alcohol and allowed to air-dry for 5 min. Hand pressure was applied to ensure uniform contact. Samples were left on the skin for a dwell time of 5 min before peeling. Peel testing was conducted at a 90° peel angle using a precision force gauge gripper (G1008, Mark-10) with unbacked samples. All measurements were performed in a climate-controlled laboratory at room temperature. To prevent skin irritation or altered surface energy, a 10 min recovery interval was enforced between consecutive trials on the same skin location.

Optical images were captured using a light photobox (Puluz Technology Ltd., Shenzhen, China). The impedance spectra of the PDE and Ag/AgCl electrodes were obtained using electrochemical impedance spectroscopy (Model Interface 1010E, Gamry Instruments, Warminster, PA, USA). The frequency range was 10^−1^ to 10^4^ Hz, and the estimated Z was selected as 10^3^ for the user interface menu of the EIS apparatus.

### 2.4. Biopotential Signal Detection

ECG and EMG signals were acquired with an Arduino Uno microcontroller. Cardiac and heart-rate monitoring used an AD8232 single-lead front end configured for a 0.5–40 Hz passband. The 0.5 Hz high-pass corner removes the DC offset and slow baseline drift, and the 40 Hz low-pass corner lies below the mains frequency, so no notch filter was used. Signals were digitised at 401.8 Hz, with a sample interval of 2.489 ms, which is five times the Nyquist rate for the 40 Hz upper corner. No digital filtering was applied before the RMS calculation. PDE electrodes with a 3 cm × 3 cm (9 cm^2^) effective contact area were used for both ECG and EMG; the commercial Ag/AgCl gel electrodes used for comparison have a conductive gel area of 2.25 cm^2^.

Baseline noise was quantified from the isoelectric TP segments of five consecutive cardiac cycles, with the P wave, the QRS complex and the T wave excluded from the summation:

(1) $$V_{RMS,noise}=\sqrt{\frac{1}{N}\sum_{i=1}^{N}v^{2}\left(t_{i}\right)},t_{i}\in \overset{5}{\underset{j=1}{⋃}}{TP}_{j}$$

where TP*_j_* is the isoelectric TP segment of the *j*-th cardiac cycle, *N* is the total number of samples contained in the five segments, and *v*(*t_i_*) is the recorded potential of sample *i*.

Raw EMG signals were obtained using a MyoWare muscle sensor (Model SEN-13723; Advancer Technologies LLC, Raleigh, NC, USA). The two PDE electrodes were mounted on the forearms along the same tendon line at 2 cm distances, while the PDE reference electrodes were positioned along different tendon lines, 2 cm away from the working electrodes.

### 2.5. Sample Numbers, Replicates, and Error Bars

Tensile properties (Young’s modulus, toughness, elongation at break) and electrical conductivity were measured on *n* = 5 independent specimens per composition, prepared from five independently cast films. Peel testing on glass substrates was likewise performed on *n* = 5 independent specimens. Error bars in for these measurements denote the standard deviation (SD) across these independent samples. Cross-sectional PDMS domain sizes and FFT-derived FWHM values were extracted from three SEM images per composition (5 domains measured per image, *n* = 15 measured domains total); reported error bars represent the standard deviation across measured domains, describing microstructural dispersion within a film rather than film-to-film variability.

Cyclic tensile hysteresis (300 cycles), normalized resistance variation under cyclic strain (250 cycles), and strain-dependent normalized resistance were recorded continuously on single representative specimens. No error bars are applied to these continuous time-series data.

Skin peel force, electrode–skin impedance, ECG, and EMG signals were evaluated in a single participant (*n* = 1, co-author of this study) with *n* = 10 repeated measurements per condition acquired across seven separate days. Error bars denote the standard deviation across these repeated trials within the single individual. These metrics represent within-participant repeatability (reusability) and must not be interpreted as population-level variability. All data are reported as mean ± SD.

## 3. Results and Discussion

### 3.1. Material and Fabrication Process

**Figure 1:**
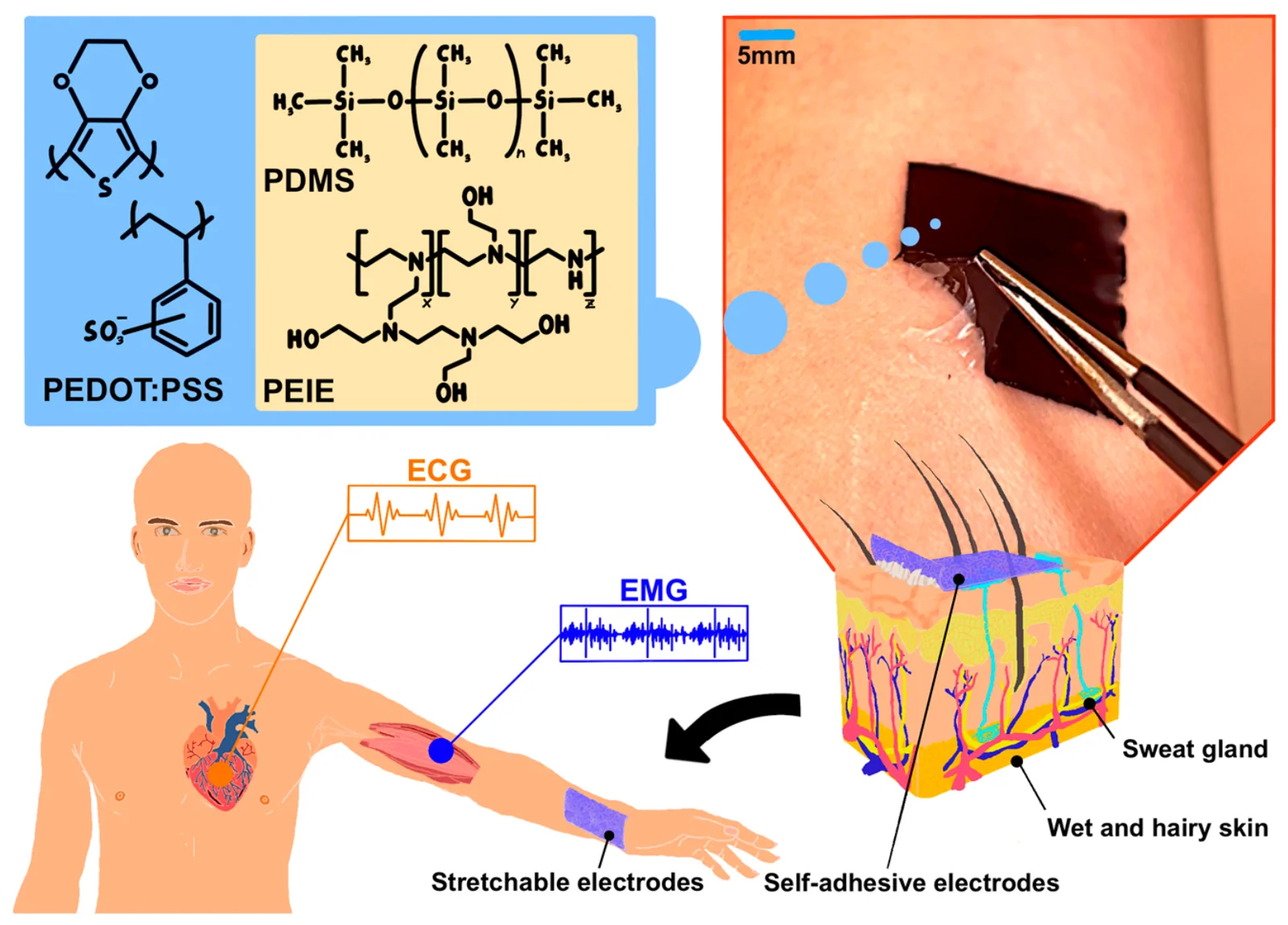
Schematic illustration of reusable and soft epidermal electrodes. The PDE electrodes consisting of PEDOT:PSS, PDMS and PEIE can be directly attached to wet and hairy epidermis. These self-adhesive, stretchable electrodes collect quality signals such as electrocardiograms and electromyograms from human beings.

Figure 1 illustrates the formation of self-adhesive films composed of PEDOT:PSS, PDMS, and PEIE, which can tightly adhere to wet and hairy skin. Uniformly emulsified films were prepared using an amphiphilic surfactant, Tween 80 [28], whose chemical structure enables the full co-dispersion of otherwise immiscible materials, such as hydrophilic PEDOT:PSS and hydrophobic PDMS (Figure S1) [39,40]. These composites have trade-off relationships between conductivity and stretchability (Figure S3) depending on the mass ratio selection; therefore, we selected 35 wt% of concentrated PEDOT:PSS for the total mass owing to less reduction in conductivity when stretchability increases. Within these PEDOT:PSS–PDMS composites, various amounts of PEIE were loaded, and the resulting formulations are designated as “PDE films” throughout this study.

### 3.2. Structural and Microscopic Characterization

**Figure 2:**
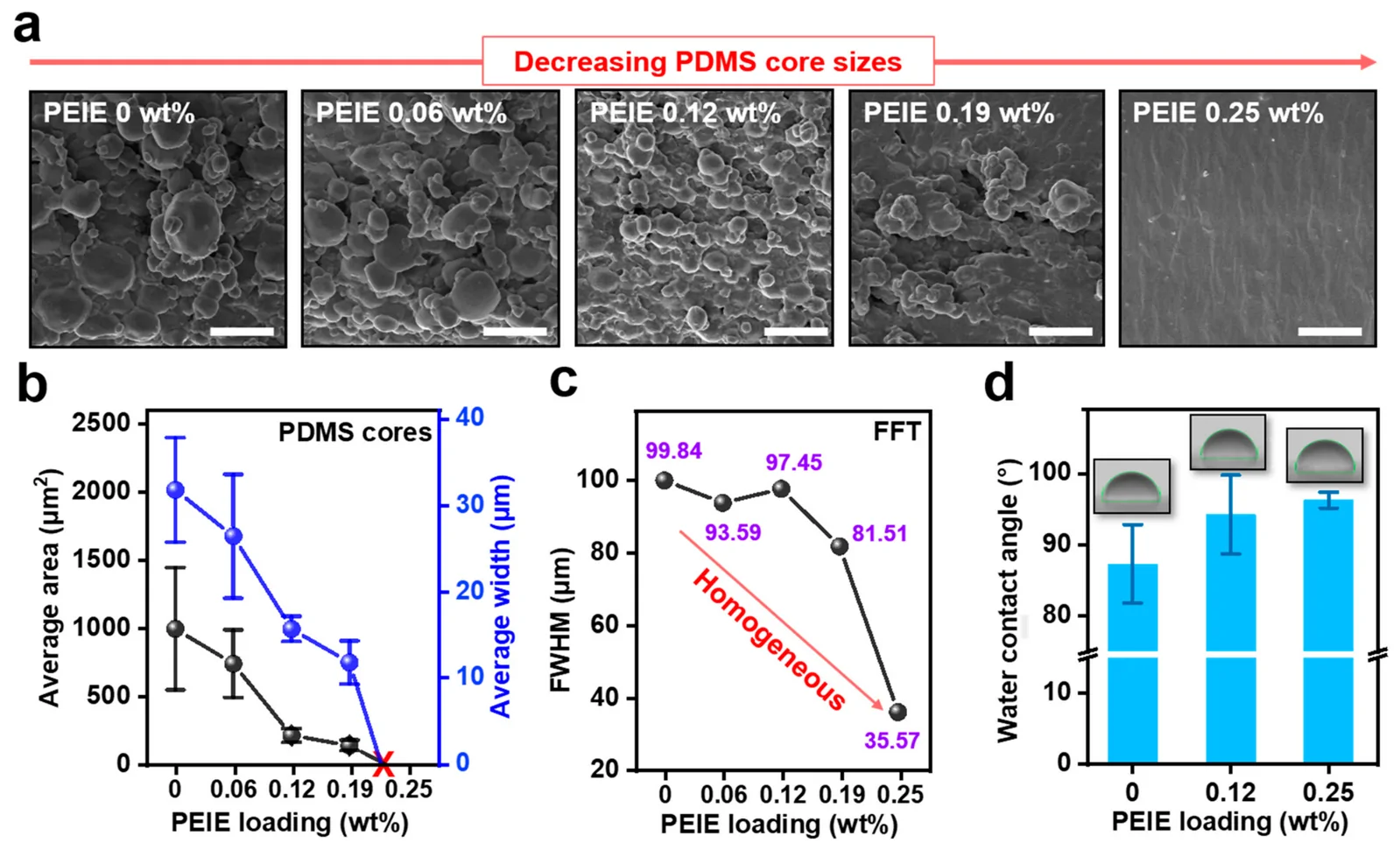
Morphological and chemical characterization of the PDE films as a function of PEIE loading. (**a**) Cross-sectional SEM images of the composite films with varying PEIE concentrations. The scale bar is 50 μm. (**b**) Quantitative analysis showing the decrease in average area and width of PDMS cores. The red cross (×) at 0.25 wt% denotes that no discrete PDMS cores could be resolved in the SEM images at this loading; no value could therefore be extracted. (**c**) FWHM values extracted from FFT images, demonstrating the enhanced structural homogeneity of the films. (**d**) Water contact angles of the films. The inset shows optical images of water droplets on the respective surfaces (0, 0.12, 0.25 wt%).

The microstructures of the PDE films were characterized using scanning electron microscopy (SEM) and fast Fourier transform (FFT) analysis. As shown in Figure S4, the composites formed microscale PDMS cores (~50 μm) in the absence of PEIE. Upon introducing different PEIE loads per 10 g of PDMS, a distinct decrease in the PDMS core size was observed in the cross-sectional SEM images (Figure 2a,b and Figure S6). Moreover, the decrease in the full width at half maximum (FWHM) of the brightness values [41] with increasing PEIE loading confirms the enhanced homogeneity of the films (Figure 2c, Figures S8 and S9), and the surface SEM images (Figure S7) are consistent with these internal structural findings.

X-ray photoelectron spectroscopy (XPS) analysis (Figure S10) further confirms the increased intensity of the O1s, C1s, and Si2p peaks in surface PDE films when PEIE loading rises, where PEDOT:PSS and PEIE contribute to the O1s and C1s peaks [42] and PDMS contributes to all three peaks [43]. The homogeneous surface morphology also endows the films with a water-repellent nature, as verified by water contact angle measurements (WCA) [28,44] (Figure 2d). Simple light-bulb experiments further demonstrated the water tolerance of the PDE films, confirming that they maintained their conductivity even in underwater environments (Figure S12).

These characteristics align with PEIE inhibiting the curing of the PDMS matrix. Amine additives are reported to coordinate with platinum hydrosilylation catalysts via nitrogen lone pairs, thereby retarding the cure of addition-cured silicones [37,45] (Figure S5). Because the platinum catalyst drives the addition of Si–H across vinyl groups to form Si–CH_2_–CH_2_–Si crosslinks, reduced catalyst activity yields a less fully formed network. Our mechanical data support this mechanism. Applying the affine model of rubber elasticity ($\nu_{e}=E/3RT$) to the measured Young’s moduli [46] yields effective crosslink densities ($\nu_{e}$) that drop from $362\pm 55{\;\mathrm{mol}\;m}^{-3}$ without PEIE to $87\pm 54{\;\mathrm{mol}\;m}^{-3}$ at 0.25 wt% (Figure S19). This value serves as a relative metric for comparison across compositions rather than an absolute network density. While it does not independently prove the catalyst-coordination mechanism—since any process that slows network formation would yield the same trend—this reduced crosslink density directly accounts for our observed performance: a low-modulus, highly stretchable matrix in which conductive PEDOT:PSS pathways remain intact during deformation. As illustrated schematically in Figure S18, we propose that in the absence of PEIE, the large, rigid PDMS domains disrupt the PEDOT:PSS conductive pathways upon stretching (“Easily broken path”), whereas the more homogeneous architecture of the PDE film leaves those pathways intact under strain (“Hardly broken path”). Figure S18 is a schematic of this proposal rather than an observation. Our SEM images resolve the PDMS domains, but they do not distinguish the conductive PEDOT:PSS phase from the insulating matrix, so we do not claim to have imaged the conductive network.

### 3.3. Mechanical and Electrical Properties

**Figure 3:**
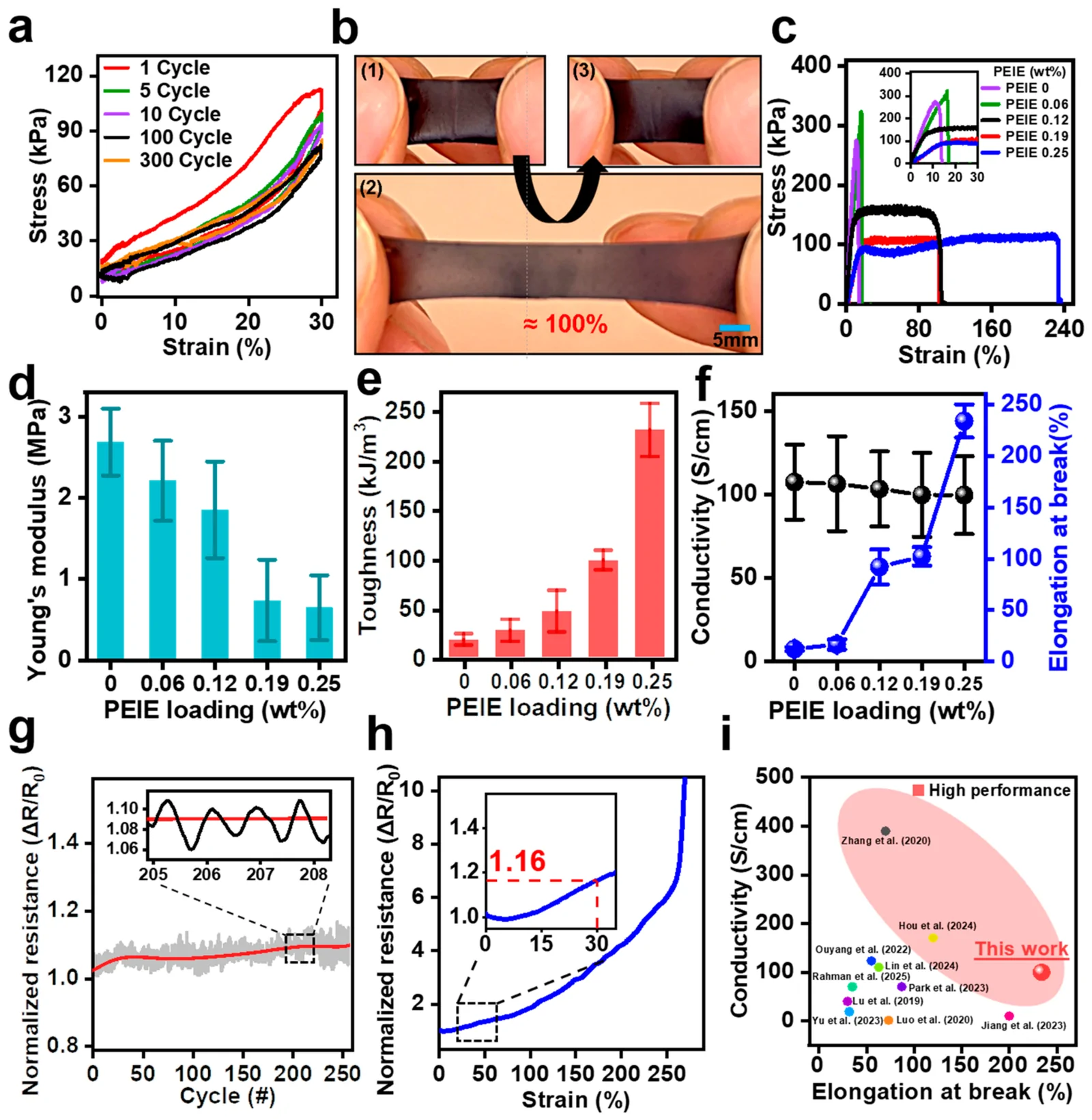
Mechanical and electrical properties of the PDE films. (**a**) Cyclic tensile stress–strain curves (hysteresis loops) of the PDE film under 30% strain for up to 300 cycles. (**b**) Photographic images of a single stretching/releasing cycle of the film up to approximately 100% strain. The numbers denote the sequence of the cycle: (1) the film in its initial, unstrained state; (2) the film uniaxially stretched to ≈100% strain; and (3) the film after release. The curved arrow indicates the stretching/releasing step from (1) to (3). The scale bar is 5 mm. (**c**) Tensile stress–strain curves of the PDE films as a function of PEIE loading volume. The inset shows that the PDE films with 0 and 0.06 wt% loading fail before reaching 20% strain. (**d**,**e**) Quantitative analysis of the (**d**) Young’s modulus and (**e**) toughness values derived from the respective stress–strain curves as a function of PEIE loading. (**f**) Electrical conductivity and elongation at break plotted against PEIE loading volume, displaying an enhanced stretchability without sacrificing conductivity. (**g**) Normalized resistance variation (Δ*R/R*_0_) over 250 stretching/releasing cycles at 30% strain. The grey trace is the continuously recorded raw signal, and the red line is a trend line indicating the overall drift in resistance. The inset presents the raw signal (black) resolved over the 205th to 208th cycles, together with the corresponding trend line (red). (**h**) Normalized resistance changes as a function of tensile strain until mechanical failure. The inset highlights a low resistance change of 1.16 at a daily-maximum strain of 30%. (**i**) Comparative plot of electrical conductivity versus elongation at break, evaluating the performance of the PDE film against previously reported PEDOT:PSS-based conductive films. Each data point is labelled with the first author and publication year of the corresponding study [10,22,23,28,49,50,51,52,53,54]; the colours distinguish individual points and carry no additional meaning. The shaded region denotes the high-performance regime. (note: the PEIE loading was fixed at 0.25 wt% for panels (**a**,**b**,**g**–**i**)).

The PDE films exhibited stable hysteresis loops over 300 cycles at 30% strain (Figure 3a), indicating that the film tolerates repeated loading over the range tested. Furthermore, no visible physical damage or plastic deformation was detected after a high-strain (~100%) stretching cycle (Figure 3b). At the PEIE loading of 0.25 wt%, an elongation at break of 234% was measured using a tensile tester (Figure 3c), where the Young’s modulus (~649 kPa) and toughness (~231 kJ/m^3^) were calculated from the stress–strain curve (Figure 3d,e). These mechanical properties, including a low tissue-like Young’s modulus (<1 MPa) and high stretchability (>230%), are crucial for maintaining conformal and stable adhesion to the skin [47].

Importantly, high conductivity (~100 S/cm) and a large elongation at break (~234%) were concurrently achieved at this 0.25 wt% PEIE loading (Figure 3f). However, excessive PEIE loading (>0.25 wt%) severely hindered the complete curing of the film because of steric hindrance and over-coordination of the abundant amine groups [37]. Therefore, 0.25 wt% was the highest loading that still cured completely, and this was the reason for going up to that limit. The electrical behavior under dynamic strain was further evaluated. Minimal fluctuations in the normalized resistance under 30% strain over the 250th stretching/releasing cycle were observed (Figure 3g). Even under the maximum strain typically experienced by human skin during daily activities (~30%) [48], a highly stable normalized resistance change ($\Delta R/R_{0}$) of only 1.16 was recorded (Figure 3h). This negligible resistance variation demonstrates the outstanding capability of the material to serve as a stable epidermal electrode. Moreover, the PDE films outperformed previously reported PEDOT:PSS-based conductive films [10,22,23,28,49,50,51,52,53,54] (Figure 3i).

### 3.4. Conformability and Adhesive Properties

**Figure 4:**
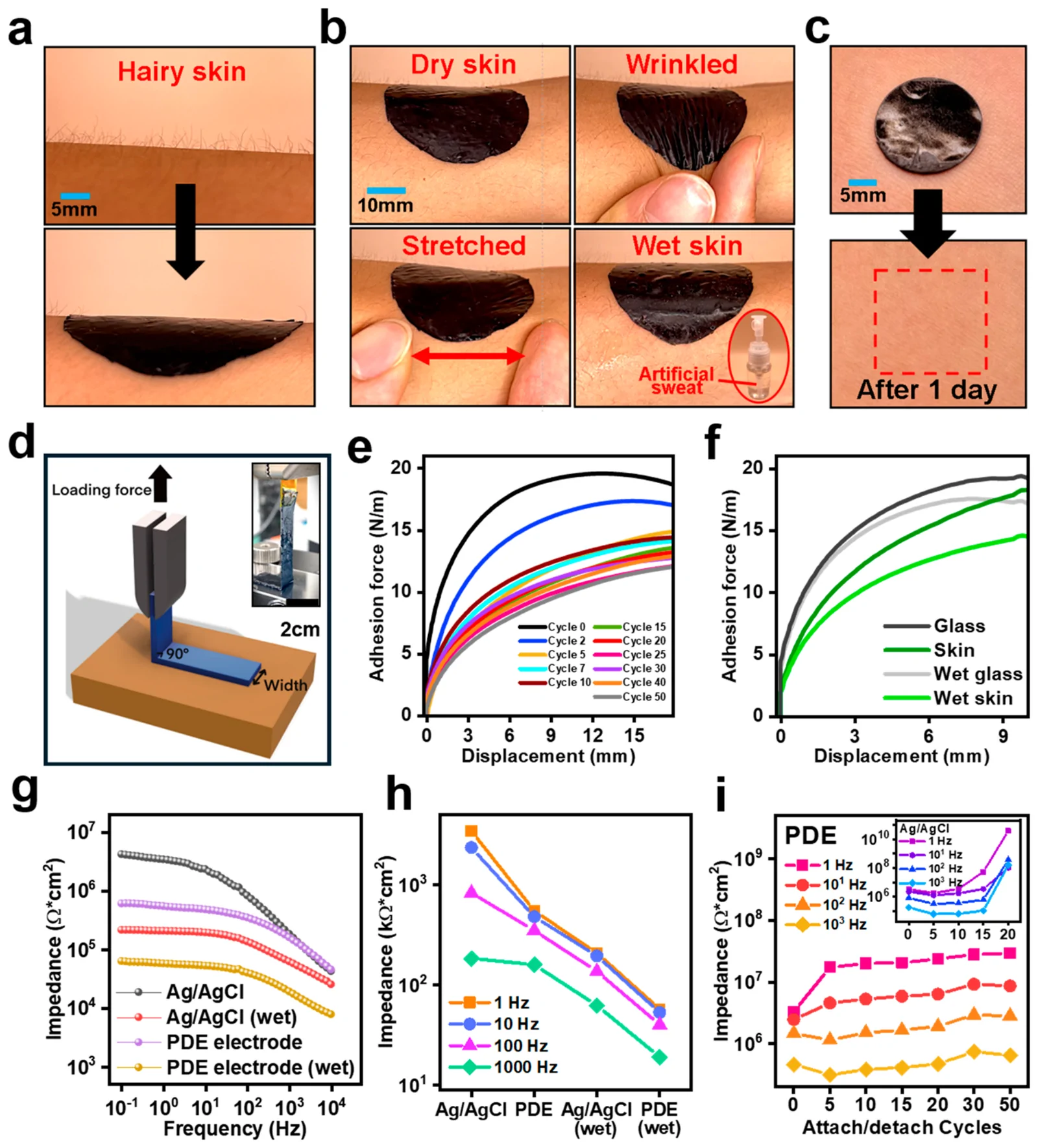
Conformability, short-term skin tolerance, and adhesion capabilities of the PDE electrodes. (**a**,**b**) Photographic images showcasing the conformability of the PDE films on (**a**) hairy skin and (**b**) various challenging skin states (dry, wrinkled, stretched, and wet skin). (**c**) Short-term skin tolerance evaluation of the PDE film on human skin, demonstrating excellent skin friendliness without any observable irritation or redness after 24 h of continuous attachment. (**d**) Schematic illustration of the 90° peel adhesion test configuration. The inset displays an optical image of the actual peel-testing experiment setup. (**e**) Cyclic peel adhesion force profiles on a glass substrate measured over 50 consecutive attachment/detachment cycles, highlighting the durable self-adhesiveness of the film. (**f**) Comparison of the maximum peel adhesion forces of the PDE film evaluated on diverse substrates under dry and wet conditions (glass, skin, wet glass, and wet skin). (**g**,**h**) Impedance spectra measured on human skin comparing the PDE electrode with a commercial Ag/AgCl gel electrode under dry and wet conditions, presented as (**g**) a continuous frequency spectrum and (**h**) a quantitative comparison at specific frequencies (1, 10, 100, and 1000 Hz). (**i**) Skin–electrode impedance of the PDE and Ag/AgCl gel electrodes tracked at selected frequencies over 50 consecutive attachment/detachment cycles. The PDE electrode shows negligible impedance variation across the full series; the inset shows the rapid degradation of the commercial Ag/AgCl gel electrode, which became unmeasurable beyond 20 cycles (note: artificial sweat was simulated using a 100 mM NaCl solution for panels (**b**,**f**–**h**)).

The PDE films exhibited outstanding compliance and conformability to diverse and challenging skin conditions, such as hairy, dry, wrinkled, stretched, and wet skin surfaces (Figure 4a,b). Although commercial Ag/AgCl gel electrodes can induce skin irritation or rashes during prolonged use [55], the PDE films, consisting entirely of organic materials, caused no observable skin irritation, even after a full day of continuous attachment (Figure 4c). The adhesion forces of the PDE films were quantitatively evaluated using the 90° peel test method (Figure 4d). Figure 4e shows the excellent adhesion abilities of the PDE films, which maintained robust adhesion forces over 50 consecutive attachment/detachment cycles. The adhesion forces on several substrates, including glass, skin, wet glass, and wet skin, were also measured (Figure 4f). Because peel force in viscoelastic adhesives depends strongly on displacement rate, the values reported in Figure 4e,f can be compared only to literature data obtained at similar rates. Direct quantitative comparisons with data acquired at different peel rates, such as the 300 mm min^−1^ standard in ASTM D3330 [38], are not valid. The lower crosslink density of the PDMS phase leaves the siloxane chains more mobile, which increases the effective contact area with the substrate [56].

Electrochemical impedance spectroscopy (EIS) was performed to evaluate the contact impedance at the skin–electrode interface. Figure 4g,h demonstrate that the PDE electrodes achieved lower interfacial impedance than the Ag/AgCl gel electrodes under both dry and wet skin conditions. Reusability was analyzed by showing stable impedances over 50 attachment/detachment cycles (Figure 4i). Compared to PDE electrodes, Ag/AgCl gel electrodes exhibit a substantial increase in impedance with repeated use; consequently, impedance measurements could not be obtained beyond 20 attachment/detachment cycles (Figure S13 and Figure 4i).

### 3.5. Electrocardiography (ECG) Detection

**Figure 5:**
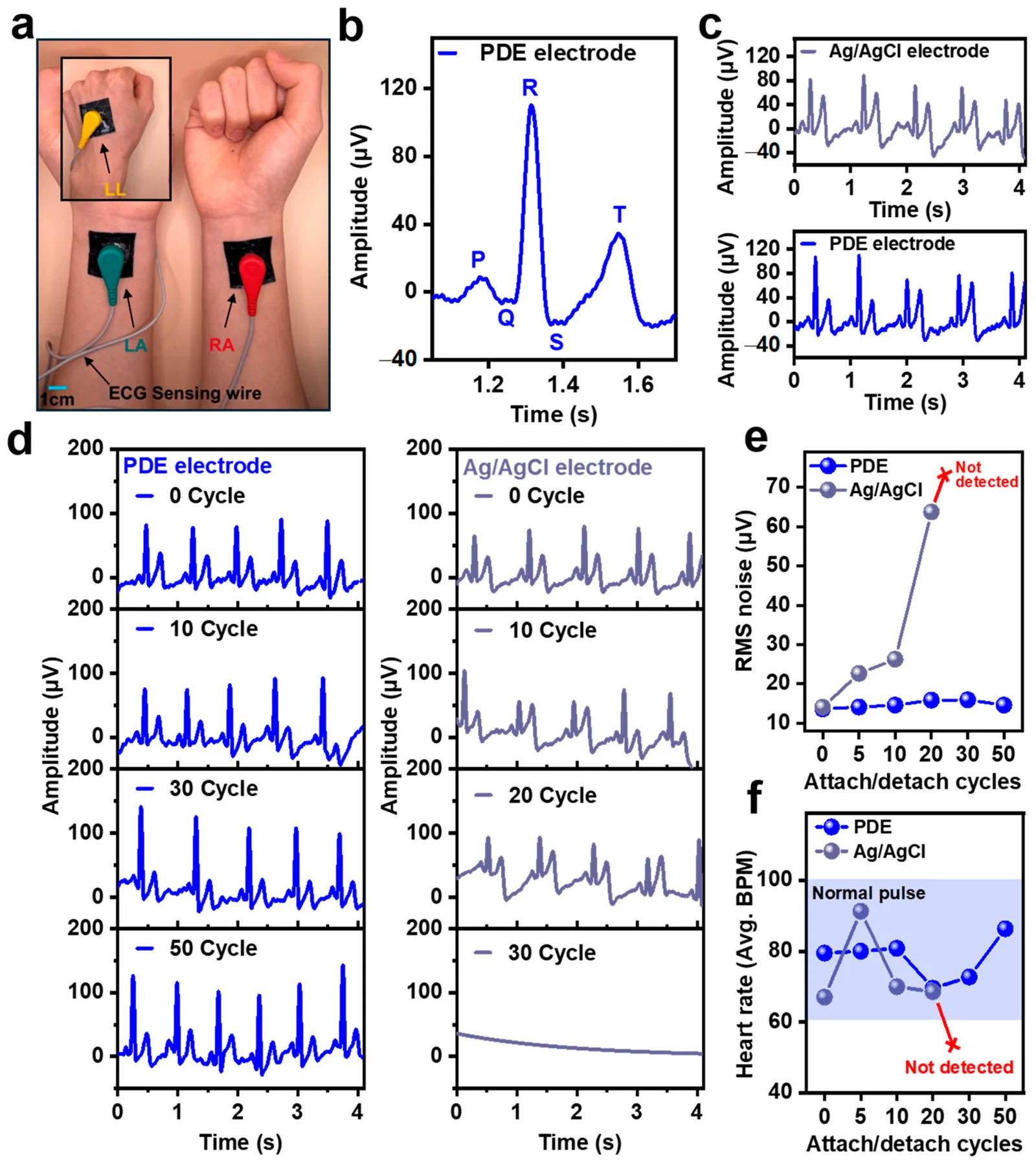
Electrocardiography (ECG) signal acquisition and reusability evaluation. (**a**) Photographic image demonstrating the ECG monitoring configuration using the self-adhesive PDE electrodes, displaying the standard lead placements on the left arm (LA), right arm (RA), and left leg/wrist (LL). (**b**) Representative high-fidelity ECG waveform captured by the PDE electrode, showcasing clearly distinguishable PQRST-waves. (**c**) Comparison of ECG signals recorded using Ag/AgCl gel electrodes (top) and the PDE electrodes (bottom). (**d**) Sequential ECG signal tracking over 50 consecutive attachment/detachment cycles, comparing the electromechanical stability of the PDE electrode (left) against the rapid degradation and signal loss of the commercial Ag/AgCl gel electrode (right). (**e**,**f**) Quantitative plots of (**e**) root-mean-square (RMS) noise levels and (**f**) extracted average heart rate profiles as a function of attachment/detachment cycles, demonstrating the robust stability of the PDE electrode up to 50 cycles, in contrast to the commercial gel electrode, which fails and becomes undetectable after 20 cycles.

Figure 5a shows an optical image illustrating the acquisition of ECG signals using the PDE electrodes, with the reference electrode placed on the rear of the left hand. The PDE electrodes successfully captured high-fidelity ECG signals with clearly distinguishable PQRST waves (Figure 5b), which are essential for diagnosing cardiovascular conditions [3,4]. The acquired ECG waveforms closely matched those obtained from commercial Ag/AgCl gel electrodes (Figure 5c). At a measured QRS peak-to-peak amplitude of 101.7 μV, the baseline RMS deviation was 12.82 μV for the PDE electrode and 13.45 μV for the gel electrode (Figure S14), corresponding to signal-to-noise ratios (SNRs) of 18.0 dB and 17.6 dB, respectively. We do not interpret this 0.42 dB difference as a performance advantage for two reasons. First, the electrodes are not area-matched (9 cm^2^ for PDE vs. 2.25 cm^2^ for Ag/AgCl). Because interfacial noise scales inversely with the square root of contact area, an interface-dominated baseline would yield an ~50% lower noise level for the four-fold larger PDE electrode, whereas the observed difference is only 4.7%. This confirms that baseline noise in our setup is governed by the recording electronics rather than the skin–electrode interface. Second, the 0.63 μV difference falls well below the single-code resolution of the analog-to-digital converter (ADC). Consequently, both electrodes provide adequate signal fidelity for ECG recording, and the meaningful distinction between them lies in how well that fidelity is preserved across repeated attachment cycles.

The primary advantage of PDE electrodes lies in their performance during repetitive ECG measurements over 50 attachment/detachment cycles. No visible degradation or baseline drift was observed in the ECG signal quality (Figure 5d and Figure S16), owing to the high retention of adhesion and the stable interfacial impedance. Conversely, conventional Ag/AgCl gel electrodes could not withstand repeated cycling and suffered from significant baseline drift and signal loss (Figure 5d,e and Figure S15). Furthermore, the heart rate information extracted from the signals recorded using PDE electrodes accurately reflected a stable, normal pulse, so the extracted heart rate remained stable across the tested cycles (Figure 5f). Within this envelope, the PDE electrode remains usable where a single-use gel electrode does not.

### 3.6. Electromyography (EMG) Detection

**Figure 6:**
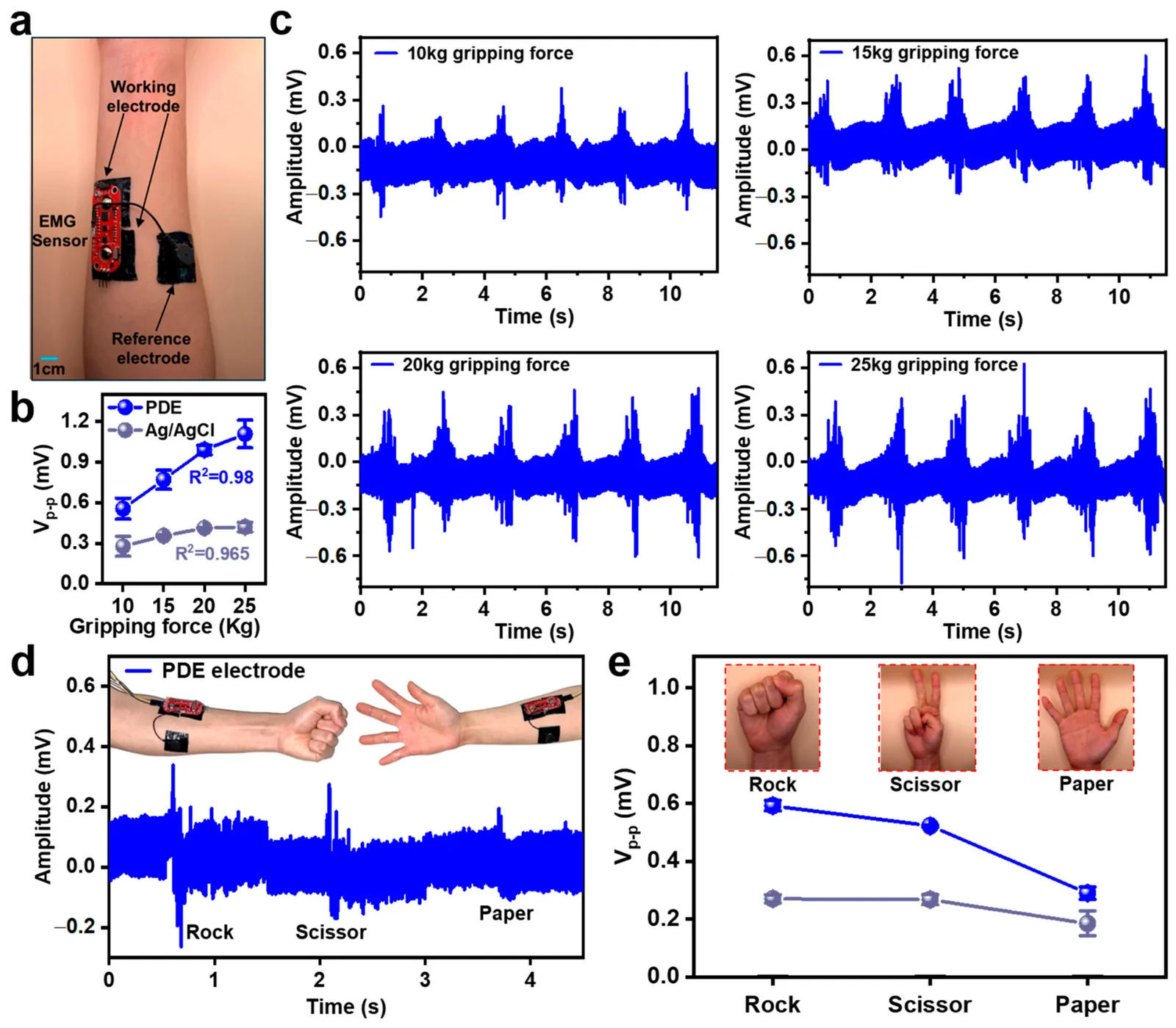
Electromyography (EMG) sensing and gesture-dependent signal amplitude for a human–machine interface. (**a**) Photographic image demonstrating the positioning of the working and reference PDE electrodes on the human forearm along the target tendon lines for EMG acquisition. (**b**) Peak-to-peak EMG amplitude versus gripping force for the PDE electrode (R^2^ = 0.980) and the Ag/AgCl gel electrode (R^2^ = 0.965), from a least-squares fit on one participant. (**c**) EMG signals recorded by the PDE electrodes at distinct gripping force levels (10, 15, 20, and 25 kg). (**d**) Continuous EMG potential profile captured by the PDE electrodes during a Rock–Paper–Scissors game, showcasing distinctive, gesture-dependent voltage amplitudes. (**e**) Peak-to-peak amplitudes for the rock, scissors and paper gestures recorded by the PDE electrode (blue) and the commercial Ag/AgCl gel electrode (grey). Error bars denote the standard deviation across repeated trials. The two electrode types differ in contact area, 9 cm^2^ against 2.25 cm^2^, so this amplitude comparison is not area-matched.

Raw EMG signals recorded at four gripping forces (Figure 6c) gave a linear relation between peak-to-peak amplitude and contraction intensity (Figure 6b). A least-squares fit at each force level on one participant gives R^2^ = 0.980 for the PDE electrode and 0.965 for the Ag/AgCl gel electrode, which would simplify the calibration of intention-detection algorithms [57]. This is a linearity measurement rather than a classification result. Temporal tracking of the EMG signals further confirmed this responsive behavior (Figure S17).

The practical utility of PDE for human–machine interfaces (HMIs) was demonstrated using a rock–paper–scissors gesture game. As shown in Figure 6d, the PDE electrodes recorded distinct changes in EMG amplitude as the hand gesture changed, and the three gestures gave separable peak-to-peak amplitudes within this session (Figure 6e). We record a larger peak-to-peak amplitude with the PDE electrodes than with the commercial Ag/AgCl electrodes, but we do not read this as a performance difference. The two are not area-matched, at 9 cm^2^ against 2.25 cm^2^, and a larger electrode samples a greater volume of underlying muscle, so a larger amplitude is expected from geometry alone. We performed no gesture classification, so no accuracy figure or confusion matrix is reported, and we present this recording as an initial feasibility demonstration. Taken together with the stability of the skin–electrode impedance and the ECG waveform across 50 attachment and detachment cycles (Figure 4i and Figure 5d), it indicates the kind of interfacial stability that continuous monitoring and machine-learning-assisted HMI systems require.

### 3.7. Scope and Limitations

All on-skin evaluations in this study—including peel force, skin–electrode impedance, ECG, and EMG—were conducted in a single participant (n=1), an author of this work, under approval from the Inha University Institutional Review Board (IRB No. 250804-2A). With a single participant, between-subject variance cannot be evaluated, and population-level claims cannot be made. Computing hypothesis tests across repeated attachment and detachment cycles would treat technical replicates from one subject as independent statistical units, resulting in pseudoreplication and artificially low p-values. Consequently, we report no inferential statistics for any on-skin measurements.

Because both electrode types were evaluated on the same participant at identical anatomical sites under identical conditions, between-subject variability was effectively controlled. Nevertheless, these findings represent single-subject feasibility data, and multi-subject trials will be required before making generalizable claims regarding comparative signal quality. Additionally, the contact areas of the electrodes were not matched ($9{\;\mathrm{cm}}^{2}$ for the PDE electrode vs. $2.25{\;\mathrm{cm}}^{2}$ for the Ag/AgCl gel electrode). Neither the baseline comparison in Figure 5e nor the impedance data in Figure 4g,h are area-normalized; all comparative evaluations are strictly restricted to the tested geometries.

The EMG demonstration is subject to the same limitations. Because we performed no gesture classification, no classification accuracy metrics or confusion matrices are reported. The three gestures were recorded during a single session with one participant with fixed electrode placement. Because EMG amplitude depends strongly on electrode position, separability at one site may not carry over to another. Reliable gesture identification would require repeated trials analyzed with an explicit classifier, reported confusion matrices, multi-participant cohorts, inter-session electrode re-application, and testing under limb motion to assess artifact robustness.

## 4. Conclusions

In summary, the incorporation of microliter quantities of PEIE played a multifunctional role in PEDOT:PSS–PDMS composites, leading to highly homogeneous films with an exceptional balance of stretchability, low Young’s modulus, and high toughness. By modulating the coordination state of the Pt catalyst, the additive tailored the polymer chain architecture to yield highly flexible siloxane networks that ensured strong skin conformability, robust self-adhesion, and low interfacial impedance. Concurrently, the electrical conductivity was maintained and the resistance of the film changed little under strain, indicating that the PEDOT:PSS conductive pathways remain continuous within the soft PDMS matrix. The practical robustness and repeated-use of the developed PDE electrodes were thoroughly shown by recording cardiac signals through 50 attachment and detachment cycles, and by resolving distinguishable EMG amplitude levels for different hand gestures in an HMI demonstration. Consequently, this soft electrode platform and its efficient additive modulation strategy offer a promising paradigm for overcoming the persistent material tradeoffs in next-generation wearable healthcare systems and soft robotics.

## Data Availability

The data presented in this study are available on request from the corresponding author.

## Supplementary Materials

The following supporting information can be downloaded at: https://www.mdpi.com/article/10.3390/mi17091066/s1, Figure S1: Role of Tween 80 as an amphiphilic surfactant within PDMS-PEDOT:PSS matrices; Figure S2: Fabrication process of reusable and soft films with varying PEIE loading; Figure S3: Trade-off relationship between conductivity and stretchability; Figure S4: SEM images of PEDOT:PSS-PDMS composites without PEIE; Figure S5: Schematics of the formation of coordination bonding between the amine group and Pt catalyst; Figure S6: Illustration of how to obtain core area and widths within SEM images; Figure S7: Surface SEM images depending on PEIE loading; Figure S8: Illustration of full-width at half maximum (FWHM) extraction from SEM images via fast Fourier transform (FFT) analysis; Figure S9: Decreasing FWHM from FFT images depending on PEIE loading; Figure S10: X-ray photoelectron spectroscopy (XPS) analysis as PEIE loading increases; Figure S11: FT-IR spectra of PDE films related to PDMS chemical bonding; Figure S12: Light bulb experiment for showing the water tolerance of the PDE composite; Figure S13: EIS analysis for measuring impedances of skin–electrode interfaces during attach/detach cycles; Figure S14: Baseline RMS deviation of the PDE and Ag/AgCl gel electrodes, and verification of the summation domain of Equation (1); Figure S15: ECG detection with pristine Ag/AgCl gel electrodes during attach/detach cycles; Figure S16: ECG detection with PDE electrodes during attach/detach cycles; Figure S17: Temporal detection of EMG signals from PDE electrodes under increasing gripping forces; Figure S18: Schematic illustration of the proposed molecular basis for the electromechanical robustness of the PDE network; Figure S19: Effective crosslink density of PDE films as a function of PEIE volume loading, estimated from tensile Young’s modulus data using the affine model of rubber elasticity.

## Abbreviations

The following abbreviations are used in this manuscript:

PEDOT:PSS	Poly(3,4-ethylenedioxythiophene) polystyrene sulfonate
PDMS	Polydimethylsiloxane
PEIE	Poly(ethyleneimine) ethoxylate
ECG	Electrocardiography
EMG	Electromyography
HMI	Human–Machine Interface
EIS	Electrochemical Impedance Spectroscopy

## References

[1] Choi S., Lee H., Ghaffari R., Hyeon T., Kim D. (2016). Recent Advances in Flexible and Stretchable Bio-Electronic Devices Integrated with Nanomaterials. Adv. Mater..

[2] Chowdhury P., Crichton C., Finster R., Whiting G., Bihar E., Kireev D. (2026). Skin Conformal Hydrogel Bioelectrodes for High-Fidelity Electrophysiology and Human-Machine Interfaces. Adv. Healthc. Mater..

[3] Hong Y., Jeong H., Cho K., Lu N., Kim D. (2019). Wearable and Implantable Devices for Cardiovascular Healthcare: From Monitoring to Therapy Based on Flexible and Stretchable Electronics. Adv. Funct. Mater..

[4] Palumbo A., Vizza P., Calabrese B., Ielpo N. (2021). Biopotential Signal Monitoring Systems in Rehabilitation: A Review. Sensors.

[5] Cheng L., Li J., Guo A., Zhang J. (2023). Recent advances in flexible noninvasive electrodes for surface electromyography acquisition. npj Flex. Electron..

[6] Lapatki B., van Dijk J., Jonas I., Zwarts M., Stegeman D. (2004). A thin, flexible multielectrode grid for high-density surface EMG. J. Appl. Physiol..

[7] Pan Y., Cui X., Song D., Hu W., Lin X., Liu N. (2024). A Stretchable and Sweat-Adhesive 3D Graphene Eutectogel Electrode for EMG Monitoring. ACS Appl. Nano Mater..

[8] Dong J., Hou J., Peng Y., Zhang Y., Liu H., Long J., Park S., Liu T., Huang Y. (2024). Breathable and Stretchable Epidermal Electronics for Health Management: Recent Advances and Challenges. Adv. Mater..

[9] Liang Y., Offenhaeusser A., Ingebrandt S., Mayer D. (2021). PEDOT:PSS-Based Bioelectronic Devices for Recording and Modulation of Electrophysiological and Biochemical Cell Signals. Adv. Healthc. Mater..

[10] Lin X., Ou Z., Wang X., Wang C., Ouyang Y., Mwakitawa I., Li F., Chen R., Yue Y., Tang J. (2024). Self-adhesive and biocompatible dry electrodes with conformal contact to skin for epidermal electrophysiology. Interdiscip. Mater..

[11] Lee E., Kim M., Lee C. (2019). Skin-Mountable Biosensors and Therapeutics: A Review. Annu. Rev. Biomed. Eng..

[12] Mazurenko I., Etienne M., Francius G., Vakulko I., Walcarius A. (2016). Macroporous carbon nanotube-carbon composite electrodes. Carbon.

[13] Tjon K., Yuan J. (2019). Impedance characterization of silver/silver chloride micro-electrodes for bio-sensing applications. Electrochim. Acta.

[14] Webb R., Bonifas A., Behnaz A., Zhang Y., Yu K., Cheng H., Shi M., Bian Z., Liu Z., Kim Y.-S. (2013). Ultrathin conformal devices for precise and continuous thermal characterization of human skin. Nat. Mater..

[15] Jaladanki C., Taxak N., Varikoti R., Bharatam P. (2015). Toxicity Originating from Thiophene Containing Drugs: Exploring the Mechanism using Quantum Chemical Methods. Chem. Res. Toxicol..

[16] Liu H., Wang L., Lin G., Feng Y. (2022). Recent progress in the fabrication of flexible materials for wearable sensors. Biomater. Sci..

[17] Park H., Kim S., Lee J., Lee I., Bontapalle S., Na Y., Sim K. (2024). Organic flexible electronics with closed-loop recycling for sustainable wearable technology. Nat. Electron..

[18] Noh E., Um H., Park H., Kim M., Kim M., Lee S., Lee H.W., Lee J., Lee B.H. (2025). Stretchable Dry Adhesives for Robust Epidermal Biosignal Sensing. Adv. Healthc. Mater..

[19] Ma H., Hou J., Xiao X., Wan R., Ge G., Zheng W., Chen C., Cao J., Wang J., Liu C. (2024). Self-healing electrical bioadhesive interface for electrophysiology recording. J. Colloid Interface Sci..

[20] Meziane N., Webster J., Attari M., Nimunkar A. (2013). Dry electrodes for electrocardiography. Physiol. Meas..

[21] Meng X., Mo L., Han S., Zhao J., Pan Y., Wang F., Fang Y., Li L. (2023). Pressure-Temperature Dual-Parameter Flexible Sensors Based on Conformal Printing of Conducting Polymer PEDOT:PSS on Microstructured Substrate. Adv. Mater. Interfaces.

[22] Cao J., Yang X., Rao J., Mitriashkin A., Fan X., Chen R., Cheng H., Wang X., Goh J., Leo H.L. (2022). Stretchable and Self-Adhesive PEDOT:PSS Blend with High Sweat Tolerance as Conformal Biopotential Dry Electrodes. ACS Appl. Mater. Interfaces.

[23] Zhang L., Kumar K., He H., Cai C., He X., Gao H., Yue S., Li C., Seet R.C.-S., Ren H. (2020). Fully organic compliant dry electrodes self-adhesive to skin for long-term motion-robust epidermal biopotential monitoring. Nat. Commun..

[24] Geerligs M., van Breemen L., Peters G., Ackermans P., Baaijens F., Oomens C. (2011). In vitro indentation to determine the mechanical properties of epidermis. J. Biomech..

[25] Feng X., Li G., Ramier A., Eltony A., Yun S. (2022). In vivo stiffness measurement of epidermis, dermis, and hypodermis using broadband Rayleigh-wave optical coherence elastography. Acta Biomater..

[26] Chun S., Kim D., Baik S., Lee H., Lee J., Bhang S., Pang C. (2018). Conductive and Stretchable Adhesive Electronics with Miniaturized Octopus-Like Suckers against Dry/Wet Skin for Biosignal Monitoring. Adv. Funct. Mater..

[27] Kim T., Park J., Sohn J., Cho D., Jeon S. (2016). Bioinspired, Highly Stretchable, and Conductive Dry Adhesives Based on 1D-2D Hybrid Carbon Nanocomposites for All-in-One ECG Electrodes. ACS Nano.

[28] Park H., Na M., Shin D., Kim D., Kim E., Kim S., Lee D., Sim K. (2023). A skin-friendly soft strain sensor with direct skin adhesion enabled by using a non-toxic surfactant. J. Mater. Chem. C.

[29] Carlborg C., Haraldsson T., Cornaglia M., Stemme G., van der Wijngaart W. (2010). A High-Yield Process for 3-D Large-Scale Integrated Microfluidic Networks in PDMS. J. Microelectromech. Syst..

[30] Creton C., Ciccotti M. (2016). Fracture and adhesion of soft materials: A review. Rep. Prog. Phys..

[31] Yang S., Liu C., Tang L., Shang J., Zhang J., Jiang X. (2024). Highly Adhesive and Stretchable Epidermal Electrode for Bimodal Recording Patch. ACS Appl. Mater. Interfaces.

[32] Savagatrup S., Chan E., Renteria-Garcia S., Printz A., Zaretski A., O’Connor T., Rodriquez D., Valle E., Lipomi D.J. (2015). Plasticization of PEDOT:PSS by Common Additives for Mechanically Robust Organic Solar Cells and Wearable Sensors. Adv. Funct. Mater..

[33] Mao Z., Wang J., Zhang J., Ohgi J., Zheng Y., Peng Y., Zhao L., Su Q., Huang W., Xu B. (2026). Fine-tuned multimodal large language model for autonomous state cognition system of shape-recognition 6-bar tensegrity integrated with flexible sensors. Microsyst. Nanoeng..

[34] Song Y., Nguyen T.H., Lee D., Kim J. (2024). Machine Learning-Enabled Environmentally Adaptable Skin-Electronic Sensor for Human Gesture Recognition. ACS Appl. Mater. Interfaces.

[35] Gao L., Yang J., Zhao Y., Zhao X., Zhou K., Zhai W., Zheng G., Dai K., Liu C., Shen C. (2025). Multilayer Bionic Tunable Strain Sensor with Mutually Non-Interfering Conductive Networks for Machine Learning-Assisted Gesture Recognition. Adv. Funct. Mater..

[36] Kadavath M.R.K., Nasor M., Imran A. (2024). Enhanced Hand Gesture Recognition with Surface Electromyogram and Machine Learning. Sensors.

[37] Jeong S., Zhang S., Hjort K., Hilborn J., Wu Z. (2016). PDMS-Based Elastomer Tuned Soft, Stretchable, and Sticky for Epidermal Electronics. Adv. Mater..

[38] (2025). Standard Test Method for Peel Adhesion of Pressure-Sensitive Tape.

[39] Hamza M., Abou-Gamra Z., Ahmed M., Medien H. (2020). The critical role of Tween 80 as a ‘green’ template on the physical properties and photocatalytic performance of TiO_2_ nanoparticles for Rhodamine B photodegradation. J. Mater. Sci. Mater. Electron..

[40] Zhang Y., Karimkhani V., Makowski B., Samaranayake G., Rowan S. (2017). Nanoemulsions and Nanolatexes Stabilized by Hydrophobically Functionalized Cellulose Nanocrystals. Macromolecules.

[41] Almohammed S., Oladapo S., Ryan K., Kholkin A., Rice J., Rodriguez B. (2016). Wettability gradient-induced alignment of peptide nanotubes as templates for biosensing applications. RSC Adv..

[42] Patidar R., McGettrick J., Garcia-Rodriguez R., Griffiths C., Lacey K., Parvazian E., Beynon D., Davies M.L., Watson T.M. (2025). Roll-to-roll slot-die coating of PTAA with PEDOT:PSS buffer layer for perovskite solar cells: Coating analysis by XPS mapping. J. Mater. Chem. A.

[43] Eon D., de Poucques L., Peignon M., Cardinaud C., Turban G., Tserepi A., Cordoyiannis G., Valamontes E., Raptis I., Gogolides E. (2002). Surface modification of Si-containing polymers during etching for bilayer lithography. Microelectron. Eng..

[44] Wu H., Wang C., Liu L., Liu Z., He J., Zhang C., Duan J. (2025). Bioinspired Stretchable Strain Sensor with High Linearity and Superhydrophobicity for Underwater Applications. Adv. Funct. Mater..

[45] Lou C., Liu E., Cheng T., Li J., Song H., Fan G., Huang L., Dong B., Liu X. (2022). Highly Stretchable and Self-Adhesive Elastomers Based on Polymer Chain Rearrangement for High-Performance Strain Sensors. ACS Omega.

[46] Alrefai A.A., Petroli A., Xu Y., Bull S.J., Geoghegan M. (2026). The Mechanical and Rheological Properties of Polydimethylsiloxane Elastomers Prepared by a Hydrosilylation Reaction. J. Appl. Polym. Sci..

[47] Yuan H., Shen Y., Zhao Y., Wang L., Zhao J., Zeng Y., Li S., Zhao Y., Miao Z., Wong J. (2026). Spraying Conformal Hydrogel Skins as Functional Platform. Adv. Funct. Mater..

[48] Oh J., Rondeau-Gagné S., Chiu Y., Chortos A., Lissel F., Wang G., Schroeder B.C., Kurosawa T., Lopez J., Katsumata T. (2016). Intrinsically stretchable and healable semiconducting polymer for organic transistors. Nature.

[49] Yu J., Tian F., Wang W., Wan R., Cao J., Chen C., Zhao D., Liu J., Zhong J., Wang F. (2023). Design of Highly Conductive, Intrinsically Stretchable, and 3D Printable PEDOT:PSS Hydrogels via PSS-Chain Engineering for Bioelectronics. Chem. Mater..

[50] Hou S., Lv D., Li Y., Li Z., Liu M., Yu X., Han Y. (2024). Highly Stretchable Self-Adhesive PEDOT:PSS Dry Electrodes for Biopotential Monitoring. ACS Appl. Polym. Mater..

[51] Lu B., Yuk H., Lin S., Jian N., Qu K., Xu J., Zhao X. (2019). Pure PEDOT:PSS hydrogels. Nat. Commun..

[52] Luo R., Li H., Du B., Zhou S., Zhu Y. (2020). A simple strategy for high stretchable, flexible and conductive polymer films based on—Blends. Org. Electron..

[53] Rahman M., Shon A., Joseph R., Pavlov A., Stefanov A., Namkoong M., Guo H., Bui D., Master R., Sharma A. (2025). Soft, stretchable conductive hydrogels for high-performance electronic implants. Sci. Adv..

[54] Jiang X., Zhou J., Zhong X., Hu Z., Hu R., Song Y., Zheng Q. (2023). Stretchable PEDOT:PSS/Li-TFSI/XSB Composite Films for Electromagnetic Interference Shielding. ACS Appl. Mater. Interfaces.

[55] Sekitani T., Yokota T., Kuribara K., Kaltenbrunner M., Fukushima T., Inoue Y., Sekino M., Isoyama T., Abe Y., Onodera H. (2016). Ultraflexible organic amplifier with biocompatible gel electrodes. Nat. Commun..

[56] Li C., Wang C., Keplinger C., Zuo J., Jin L., Sun Y., Zheng P., Cao Y., Lissel F., Linder C. (2016). A highly stretchable autonomous self-healing elastomer. Nat. Chem..

[57] Shen Z., Zhang Z., Zhang N., Li J., Zhou P., Hu F., Rong Y., Lu B., Gu G. (2022). High-Stretchability, Ultralow-Hysteresis Conducting Polymer Hydrogel Strain Sensors for Soft Machines. Adv. Mater..

