# Supplementary material for: Reusable and Soft Self-Adhesive Epidermal Electrodes for Human Skin Enabled by Functional Additives
Source: Micromachines (Basel). 2026 Sep 8;17(9):1066. doi: 10.3390/mi17091066 (PMC13609114; doi:10.3390/mi17091066)
Supplement: Supplementary file 1 [file micromachines-17-01066-s001.zip › micromachines-4525893-supplementary.pdf]

Supplementary Materials

Reusable and Soft Self-Adhesive Epidermal Electrodes for Human Skin Enabled by Functional Additives

Sungmin Bae [1], Dong-Jin Lee [2], Chuljin Hwang [2,3] and Dae Yu Kim [1,2,3,4,]*

1 Department of Electrical and Electronic Engineering, College of Engineering, Inha University, Incheon 22212, Republic of Korea; youaresm@inha.edu

2 Center for Sensor Systems, Inha University, Incheon 22212, Republic of Korea; djlee@inha.ac.kr (D.-J.L.); aki@inha.ac.kr (C.H.)

3 Department of Electrical and Computer Engineering, College of Engineering, Inha University, Incheon 22212, Republic of Korea

4 Inha Research Institute for Aerospace Medicine, Inha University, Incheon 22212, Republic of Korea

Correspondence: dyukim@inha.ac.kr

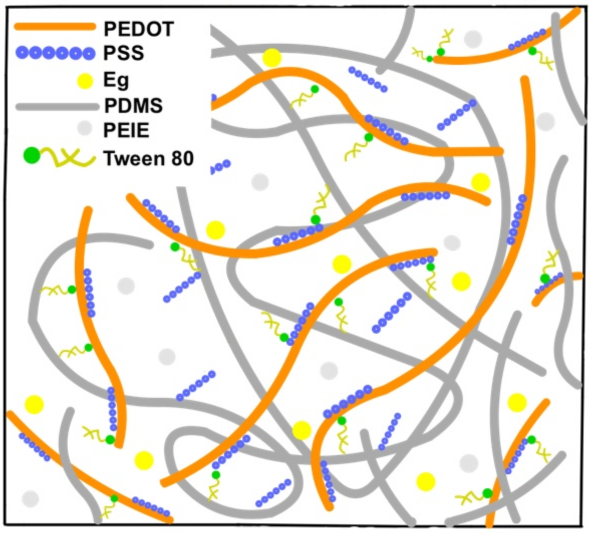

Figure S1. Role of Tween 80 as an amphiphilic surfactant within PDMS-PEDOT:PSS matrices. The hydrophilic PEDOT:PSS and hydrophobic PDMS were mixed thoroughly using Tween 80. The chemical structure of Tween 80 consists of a hydrophilic head and a hydrophobic tail; the hydrophilic head moves toward PEDOT:PSS, and the hydrophobic tail moves toward PDMS. Therefore, fully emulsified films are obtained because intrinsically non-immiscible materials are soluble in this type of amphiphilic surfactant.

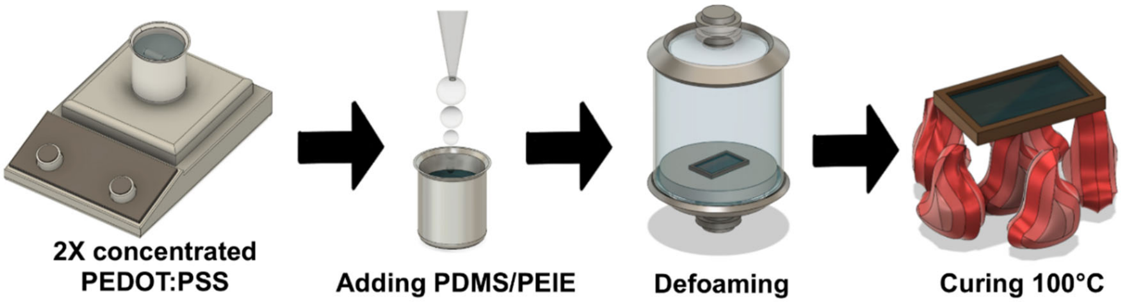

Figure S2. Fabrication process of reusable and soft films with varying PEIE loading.

The material selection and fabrication processes are described in detail in the Experimental Section.

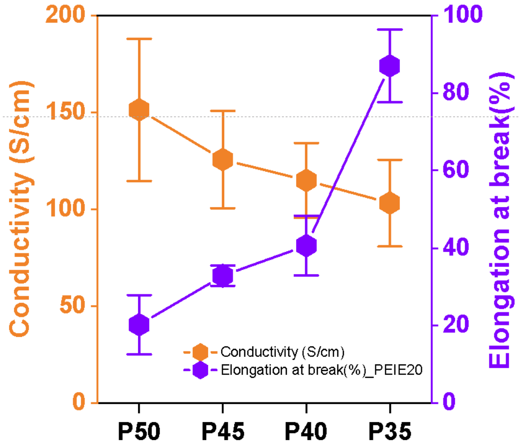

Figure S3. Trade-off relationship between conductivity and stretchability.

A varying amount of concentrated PEDOT:PSS is loaded with the same amount of PEIE loading (20 μl). When the loading of the concentrated PEDOT:PSS decreases, the electrical conductivity naturally decreases. Meanwhile, the elongation at break (%) at P35 (PEDOT:PSS 35wt% for the total mass) is significantly increased, and this ratio was selected for future research.

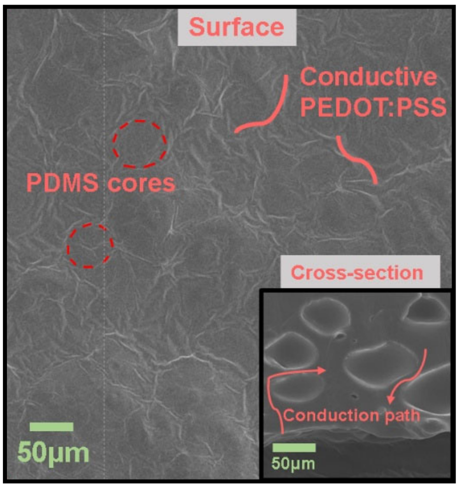

Figure S4. SEM images of PEDOT:PSS-PDMS composites without PEIE.

PDMS cores in micro units are detected in both the SEM images of the surface and cross-section. PEDOT:PSS conductive paths are present between the PDMS cores. Therefore, these composites exhibit high conductivity.

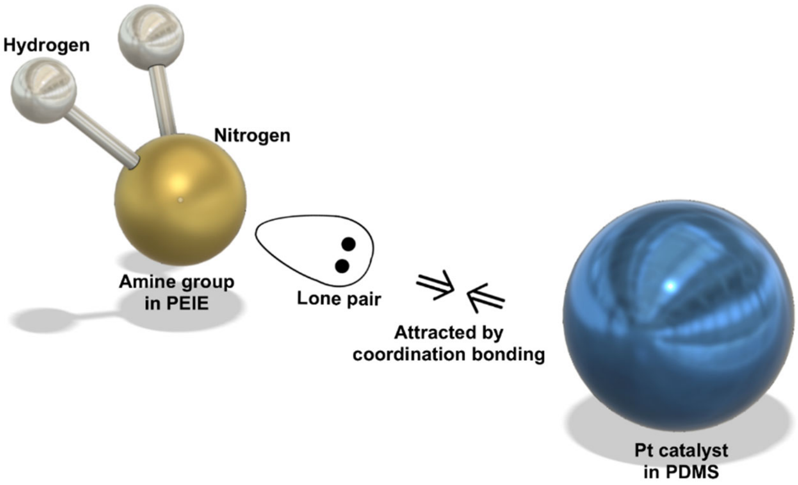

Figure S5. Schematics of the formation of coordination bonding between the amine group and Pt catalyst.

PEIE has many amine groups, and PDMS has many Pt catalysts for crosslinking the polymer chains. The amine groups consist of hydrogen and nitrogen, in which an electron-lone pair exists. This lone pair forms coordination bonds with the Pt catalyst via electrostatic attraction, leading to deactivation of the catalyst.

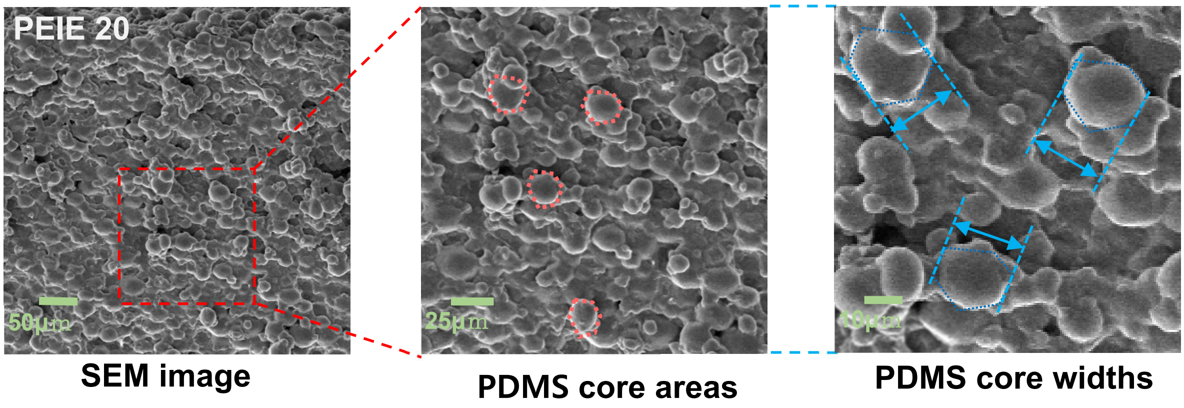

Figure S6. Illustration of how to obtain core area and widths within SEM images.

The PDMS core areas and widths are obtained from cross-sectional SEM images using ImageJ software.

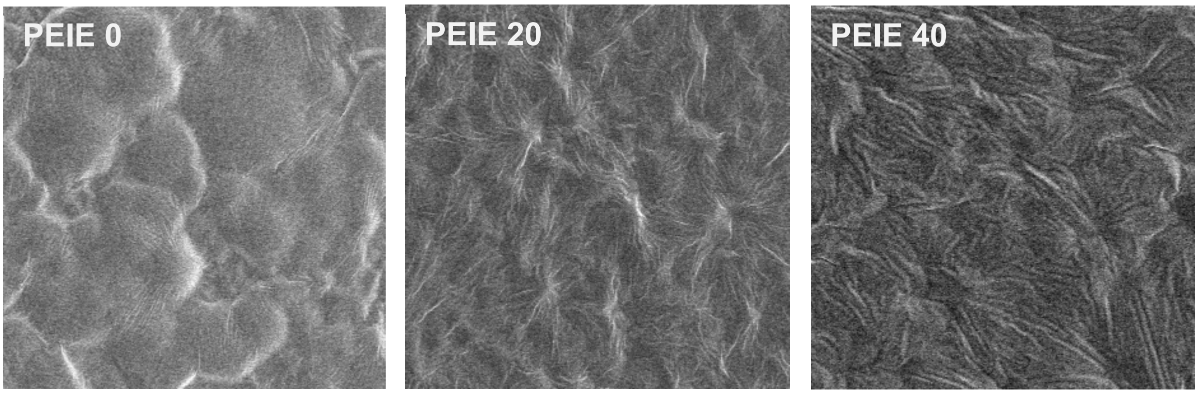

Figure S7. Surface SEM images depending on PEIE loading.

When PEIE loading is increased up to 40 μl, a more homogeneous structure is detected in the surface SEM images.

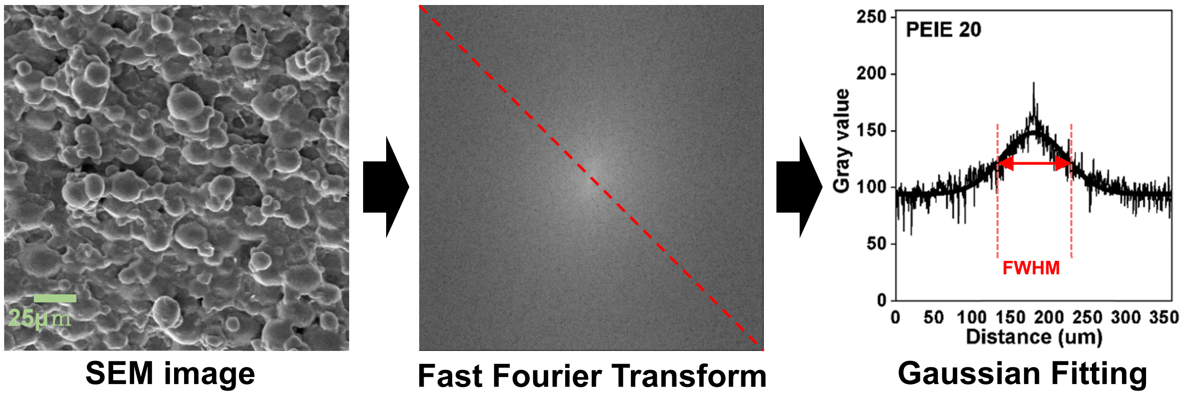

Figure S8. Illustration of full-width at half maximum (FWHM) extraction from SEM images via fast Fourier transform (FFT) analysis.

The FWHM is a representative characteristic of spectral uniformity. We found the FWHM from the line spectrum of gray scales (red dotted line) after performing FFT analysis on the cross-sectional SEM images.

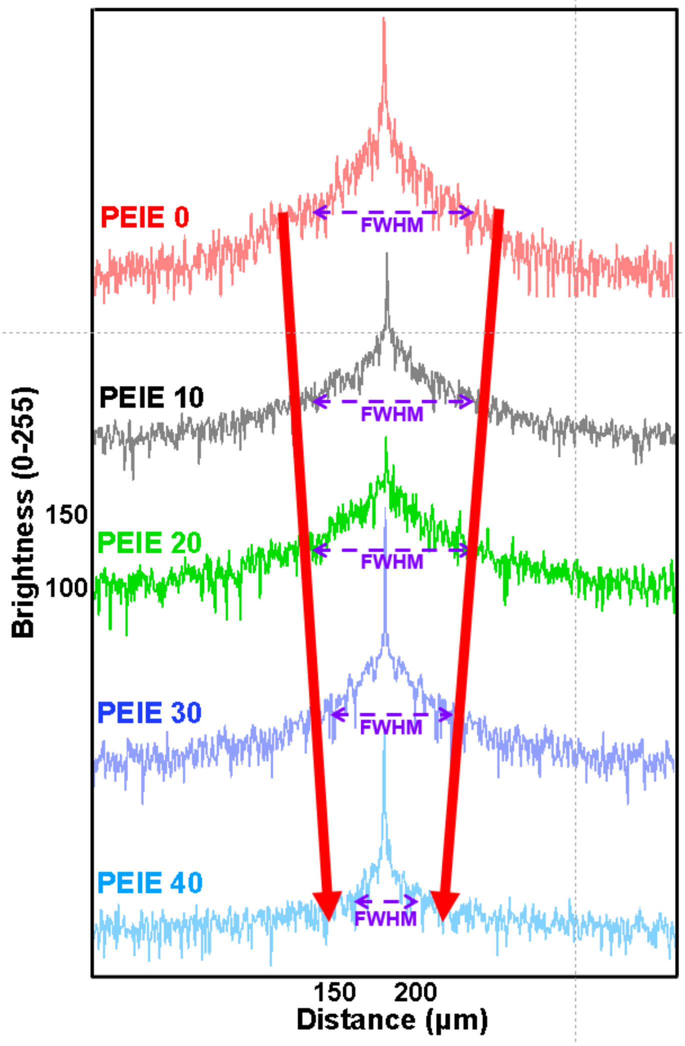

Figure S9. Decreasing FWHM from FFT images depending on PEIE loading.

A decreased FWHM of the line spectrum was detected in the FFT images, implying that the homogeneity of the film was achieved owing to the proper loading of PEIE.

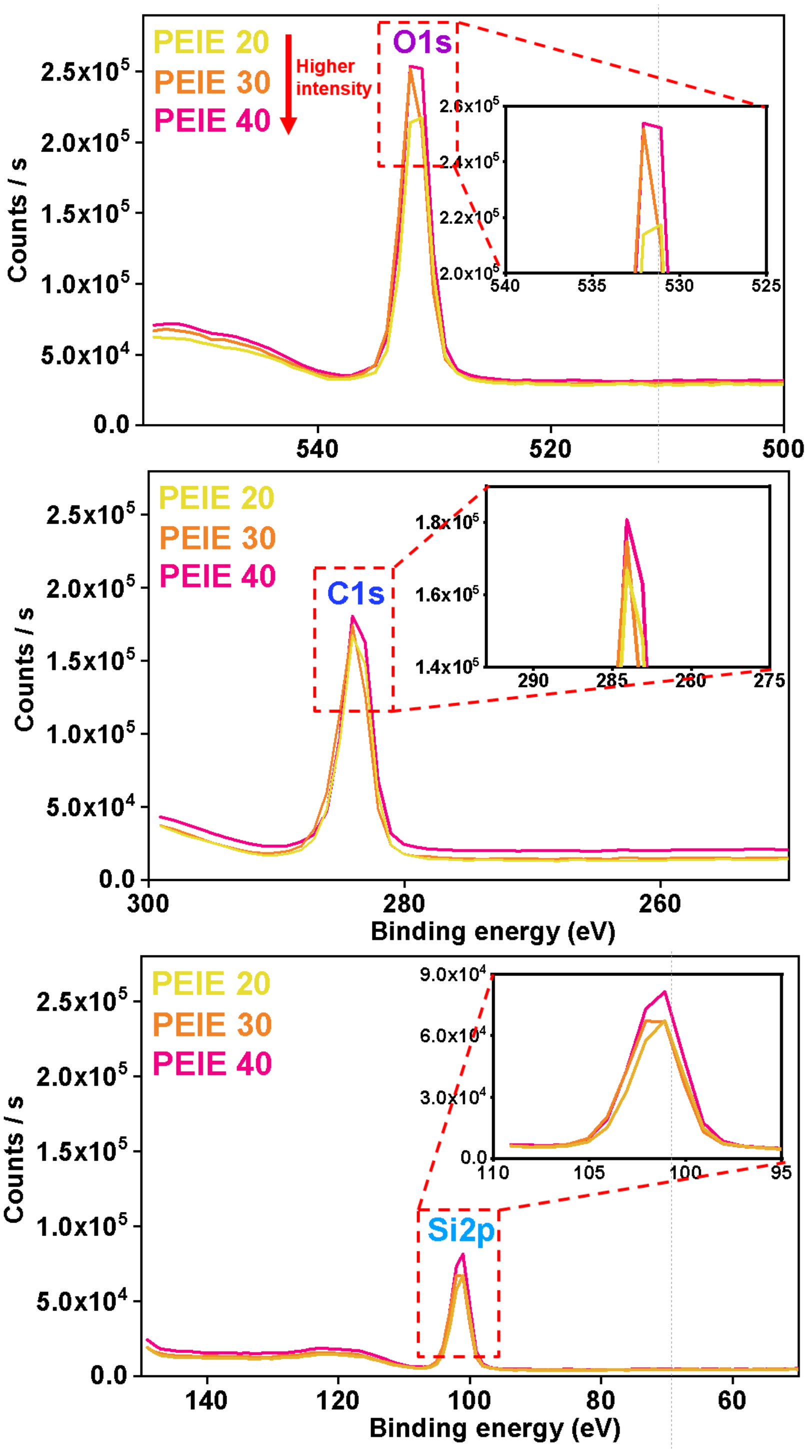

Figure S10. X-ray photoelectron spectroscopy (XPS) analysis as PEIE loading increases. We measured the intensity of the O1s, C1s, and Si2p peaks on the surfaces of the PDE films. The relatively high intensity of these peaks may be attributed to the enhanced homogeneity of the film surface when the PEIE loading increased.

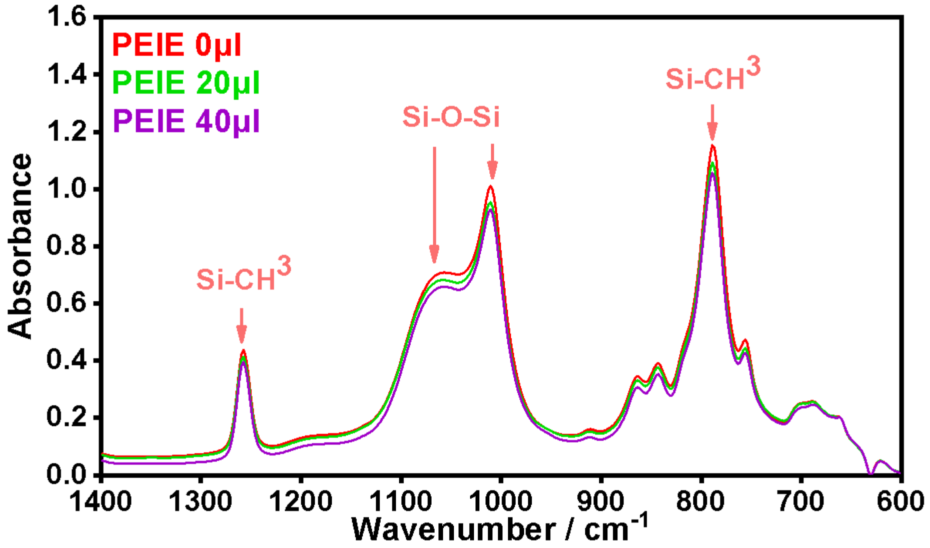

Figure S11. FT-IR spectra of PDE films related to PDMS chemical bonding.

Different absorbances were observed depending on the PEIE loading. Specifically, a reduction in the absorbance of the siloxane chain (Si-O-Si) was prominent when the PEIE loading increased. This was caused by controlling the state of the Pt catalyst to create a flexible chain of PDMS.

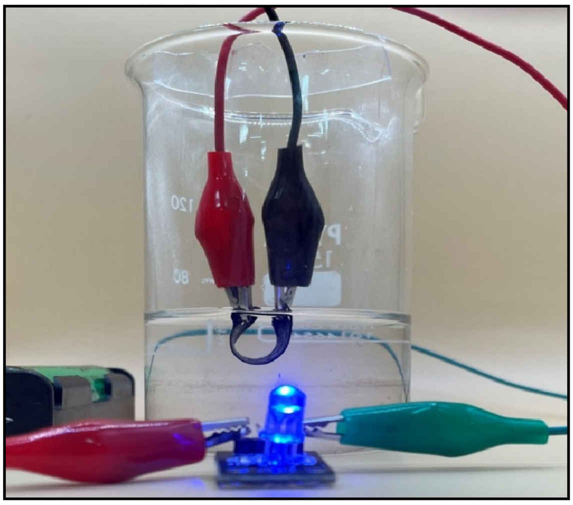

Figure S12. Light bulb experiment for showing the water tolerance of the PDE composite. The light bulbs were successfully turned on owing to the water-resistive characteristics of the PDE composite. It retained its intrinsic electrical conductivity even in underwater environments.

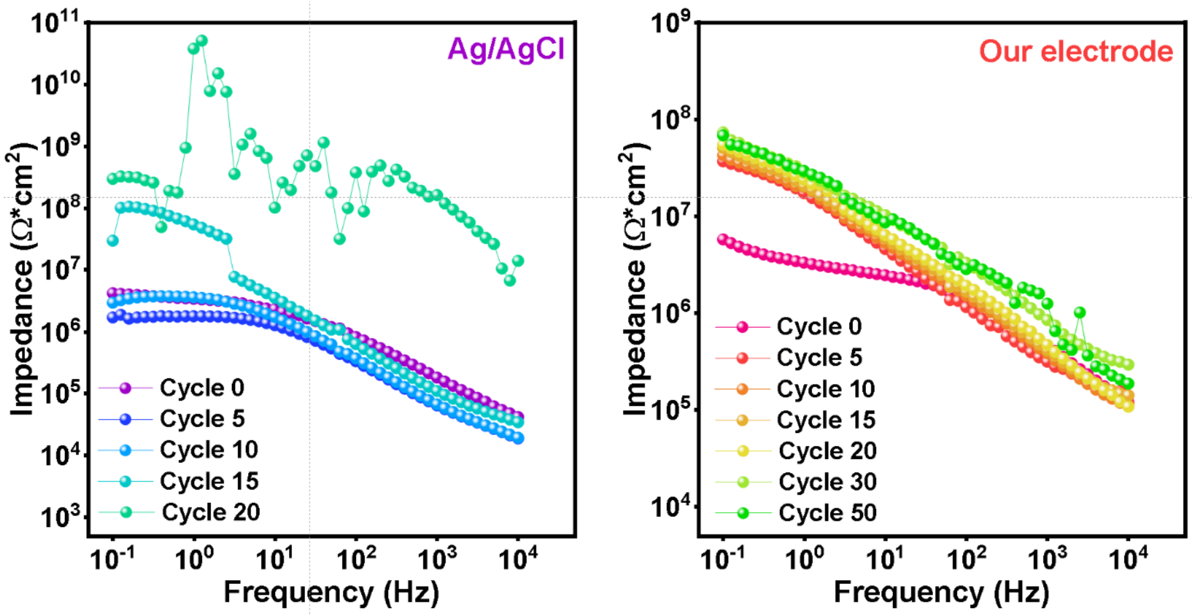

Figure S13. EIS analysis for measuring impedances of skin–electrode interfaces during attach/detach cycles.

Compared to PDE electrodes, Ag/AgCl gel electrodes cannot endure repeated attachment/detachment cycles beyond the 20th cycle, resulting in a significant baseline drift when monitoring cardiac signals, as shown in Figure S15. This demonstrates the exceptional reusability of the PDE electrodes.

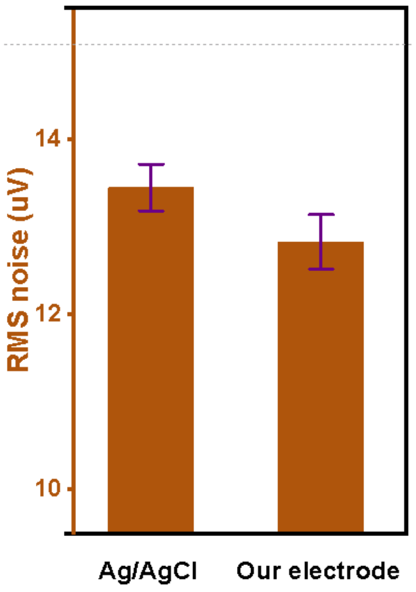

Figure S14. Baseline RMS deviation of the PDE and Ag/AgCl gel electrodes, and verification of the summation domain of Equation (1).

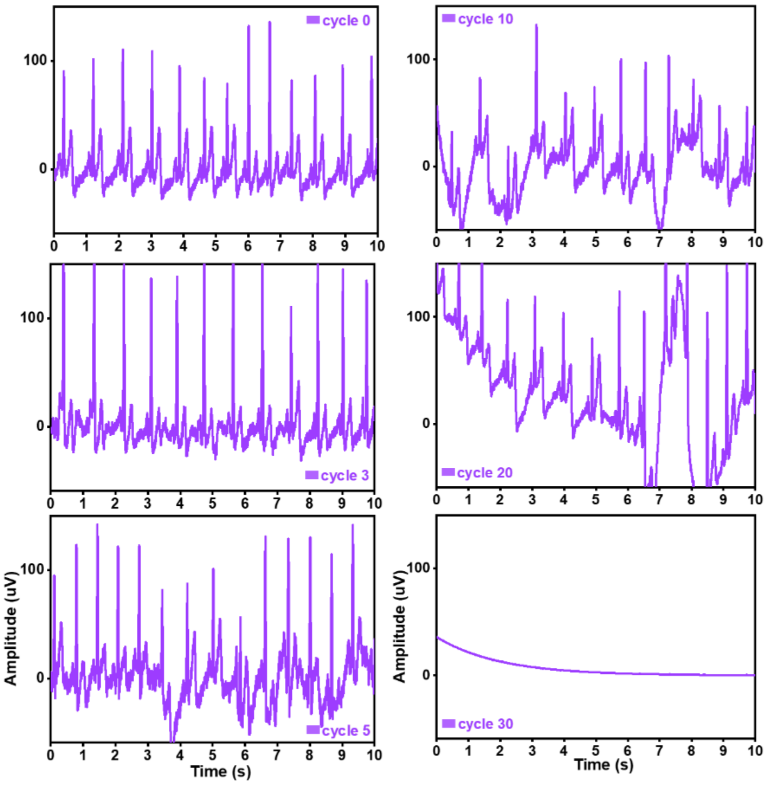

Figure S15. ECG detection with pristine Ag/AgCl gel electrodes during attach/detach cycles. These signals exhibited severe baseline drift; therefore, it was impossible to detect the ECG signals after the 30th attachment/detachment cycle.

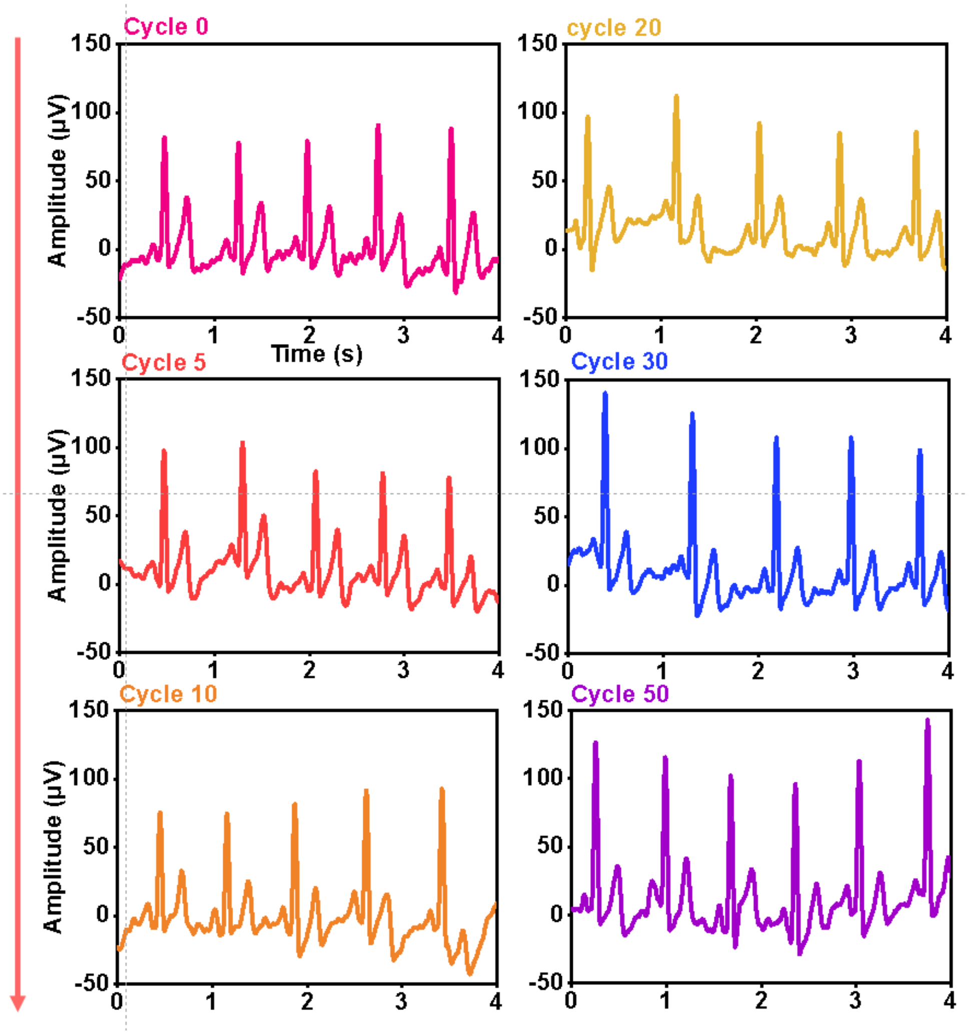

Figure S16. ECG detection with PDE electrodes during attach/detach cycles.

No significant deterioration in the ECG signals was detected in the 0th, 5th, 10th, 20th, 30th, and 50th tach/detachment cycles. The RMS noise shown in Figure 5e was calculated from the five PQRST waves in each cycle.

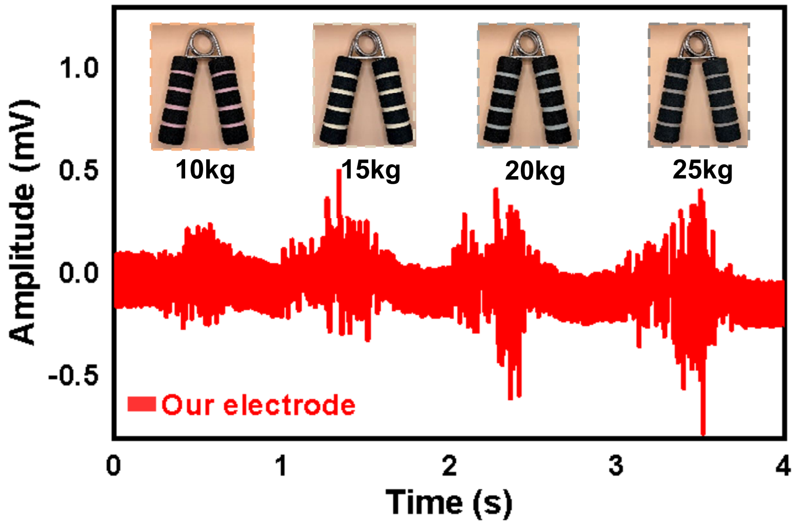

Figure S17. Temporal detection of EMG signals from PDE electrodes under increasing gripping forces.

Signals from gripping forces of 10, 15, 20, and 25 kg showed significant differences in peak-to-peak amplitudes that could be easily distinguished.

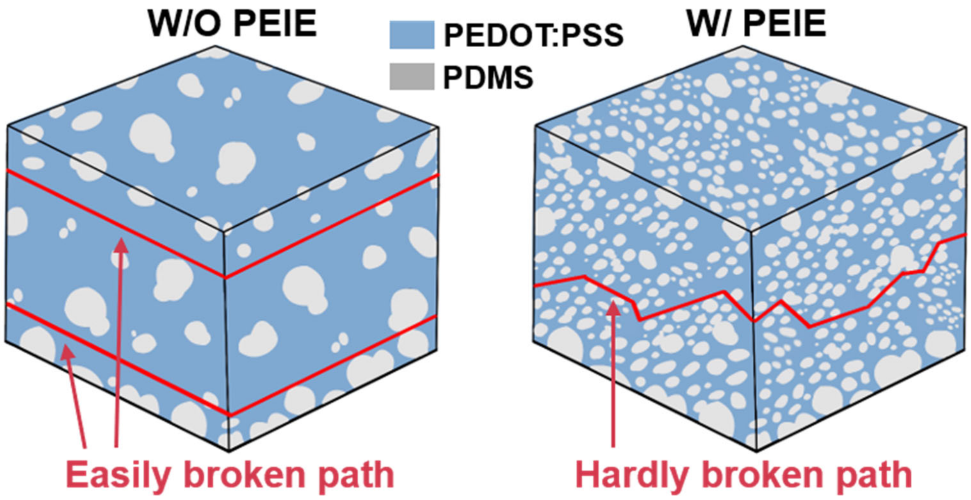

Figure S18. Schematic illustration of the proposed molecular basis for the electromechanical robustness of the PDE network. The schematic represents a hypothesis consistent with the morphological and electrical data.

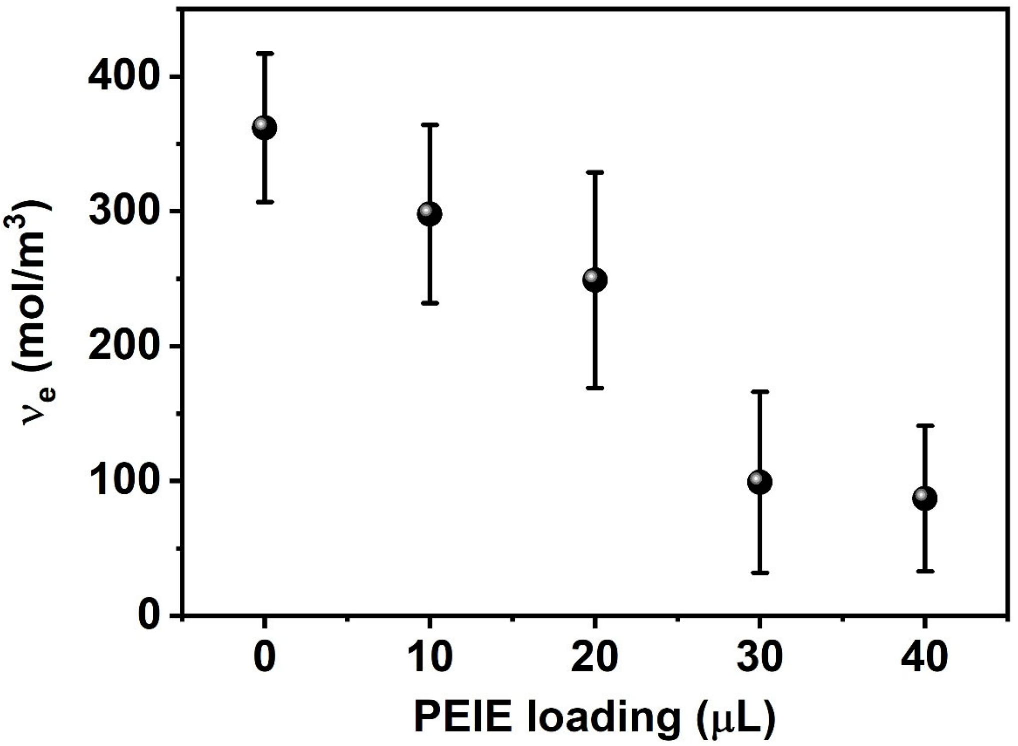

Figure S19. Effective crosslink density (υ_e) of PDE films as a function of PEIE volume loading, estimated from tensile Young's modulus data using the affine model of rubber elasticity ($\upsilon_e = E/3RT$ at T = 298 K, n = 5).
